# Propagation of activity through the cortical hierarchy and perception are determined by neural variability

**DOI:** 10.1038/s41593-023-01413-5

**Published:** 2023-08-28

**Authors:** James M. Rowland, Thijs L. van der Plas, Matthias Loidolt, Robert M. Lees, Joshua Keeling, Jonas Dehning, Thomas Akam, Viola Priesemann, Adam M. Packer

**Affiliations:** 1 Department of Physiology, Anatomy, and Genetics, University of Oxford, Oxford, UK; 2 Max Planck Institute for Dynamics and Self-Organization, Göttingen, Germany; 3 Science and Technology Facilities Council, Octopus Imaging Facility, Research Complex at Harwell, Harwell Campus, Oxfordshire, UK; 4 Department of Experimental Psychology, University of Oxford, Oxford, UK; 5 Wellcome Centre for Integrative Neuroimaging, University of Oxford, Oxford, UK; 6 Institute for the Dynamics of Complex Systems, University of Göttingen, Göttingen, Germany; 7 Present Address: Laboratory for Molecular Cell Biology, University College London, London, UK

**Keywords:** Neural circuits, Dynamical systems

## Abstract

Brains are composed of anatomically and functionally distinct regions performing specialized tasks, but regions do not operate in isolation. Orchestration of complex behaviors requires communication between brain regions, but how neural dynamics are organized to facilitate reliable transmission is not well understood. Here we studied this process directly by generating neural activity that propagates between brain regions and drives behavior, assessing how neural populations in sensory cortex cooperate to transmit information. We achieved this by imaging two densely interconnected regions—the primary and secondary somatosensory cortex (S1 and S2)—in mice while performing two-photon photostimulation of S1 neurons and assigning behavioral salience to the photostimulation. We found that the probability of perception is determined not only by the strength of the photostimulation but also by the variability of S1 neural activity. Therefore, maximizing the signal-to-noise ratio of the stimulus representation in cortex relative to the noise or variability is critical to facilitate activity propagation and perception.

## Abstract (web summary)

The authors reveal how the perception of optogenetic stimulation depends on the variability of background neural activity.

## Main

Key to the orchestration of behavior by neural systems is that information, in the form of neural activity, is reliably and accurately transmitted between anatomically distinct brain regions performing specialized tasks. Activity is transformed at each stage of its journey, and circuits often perform multiple tasks in parallel^1,2^, so it is challenging to disambiguate which facets of neural activity contribute to a specific behavior or process. In-depth analysis of cellular-resolution recordings of neural activity during sensory stimulation^3,4^ and/or well-characterized behavior^5,6^ has begun to disentangle how sensation, decisions and actions are encoded in individual brain regions. However, how sensory-related activity is structured to facilitate its journey through the brain, and how it interacts with ongoing cortical activity, is less well understood. In silico, it has been shown that the structure and variability of ongoing activity vary with internal state^7^ and can impact the classification of simple stimuli^8,9^. In vivo experiments have shown that the influence of ongoing cortical activity on sensory and behavioral responses can be of similar importance as stimulus strength^10–12^. Therefore, the relationship between sensory-related and ongoing cortical activity is crucial to understand.

One of the overarching functions of neural systems is to detect and respond to stimuli originating from the outside world. This requires reliable propagation of neural signals from sensory organs through anatomical hierarchies in the brain. Signal propagation is usually studied in psychophysical assays where stimuli of increasing strength are presented to the participant, and their detection is signaled behaviorally, while the activity of relevant brain regions is recorded to capture propagating activity^13–17^.

However, a challenge in quantifying signal propagation is that correlated activity in two regions may not reflect causal influence of one on the other but, rather, may reflect common input^18,19^. Taking causal control of neural activity by directly stimulating neurons circumvents this issue, as activity locked temporally to stimulation of a connected region likely arises from propagation. This can be achieved by driving spikes directly through stimulation of individual neurons^20–22^ or groups of neurons^23–27^. However, so far, detection of optogenetic stimulation with single-cell precision has been paired only with single-region neural recordings^24,25^, limiting signal propagation studies to within-region dynamics.

In this study, we harnessed the single-cell precision of all-optical interrogation^28^ to investigate how the collective dynamics of neural activity determine activity propagation and guide behavior. We imaged two hierarchically organized, densely interconnected and functionally well-characterized^29–32^ regions, the primary and secondary somatosensory cortex (S1 and S2), while performing two-photon (2P) optogenetic photostimulation of S1 neurons, and we trained mice to report stimulation. By recording neural activity occurring before and after photostimulation in both brain regions simultaneously, we were able to assess how behaviorally salient stimulation is propagated through anatomically distinct brain regions and interacts with their ongoing activity.

## Results

### Causally driving behavior with all-optical interrogation across cortical areas

**Fig. 1:**
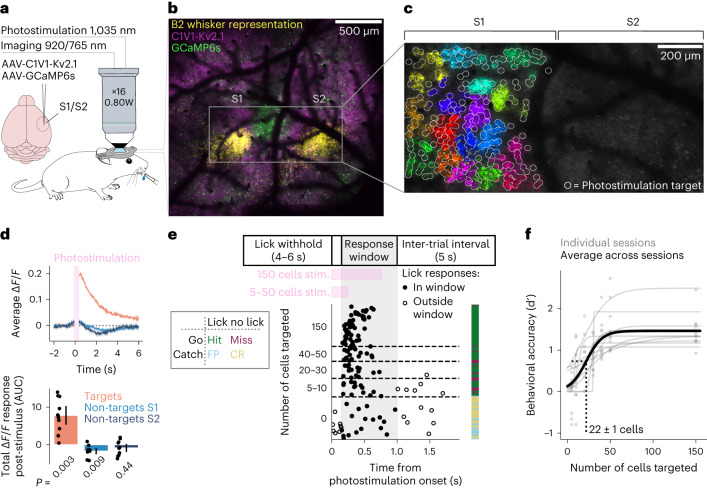
Recording neural activity in S1 and S2 during behavioral report of targeted 2P photostimulation of S1. **a**, Schematic of experimental setup. Left: viral strategy for expression of GCaMP6s and C1V1-Kv2.1-mScarlet in S1 and S2. Right: mice with a cranial window installed over S1 and S2 were head fixed under a 2P microscope. A lick spout was placed within reach of the tongue, through which the animal reported perception of photostimulation by licking and received a water reward. This figure was adapted with permission from Ethan Tyler and Lex Kravitz (Scidraw.io, 10.5281/zenodo.3925901) and Jason Keller (Scidraw.io, 10.5281/zenodo.3925969). **b**, Example imaging field of view used to localize S1 and S2 by whisker stimulation (stimulus-triggered average wide-field calcium whisker response shown in yellow; see Extended Data Fig. 1 for multiple whiskers), overlaid with aligned 2P images of GCaMP6s (green) and C1V1-Kv2.1-mScarlet (magenta) expression. **c**, Example 2P calcium imaging field of view with photostimulation targets. The intensity of each pixel is proportional to the change in fluorescence intensity post-photostimulation compared to pre-photostimulation (stimulus-triggered average; Methods); bright pixels indicate a photostimulation-induced increase in calcium activity. Pixels are color-coded based on whether they were photostimulated simultaneously. Non-targeted cells, including those in S2, are not visible because different cells would respond to repeated stimulation of the same group of targeted cells and were, therefore, averaged out. **b** and **c** show data from a representative single recording session. **d**, Top: example activity responses to photostimulation of a single recording session. Orange shows the response to photostimulation of cells directly targeted with light, averaged across cells and across trials. Light and dark blue show the response of cells not directly targeted in S1 and S2, respectively. Only trials in which photostimulation was delivered were included. Data are blanked while the photostimulation laser was on (pink bar), as this causes a large artifact unrelated to neural activity. Bottom: total Δ*F*/*F* activity post-stimulus is shown, as defined by the area under the curve (AUC) of the traces of the top panel, for all 11 recording sessions (mean ± 95% confidence interval across sessions). We tested whether each condition was significantly different from 0 (two-sided Wilcoxon signed-rank test, Bonferroni corrected). **e**, Top: timing of a single behavioral trial. Bottom left: behavioral response matrix. Bottom right: example lick raster from a single session sorted by number of cells targeted and by time within each bin. Each row of the plot shows the first lick within an individual trial. The color bar shows the outcome of the trial as defined in the behavioral response matrix. **f**, Psychometric curves showing behavioral performance (d′) as a function of the number of cells targeted by photostimulation. Each gray point is the d′ computed for a given number of cells targeted for an individual session, and each gray line is a logistic function fit for an individual session. The thick black line shows the fit for all data points across all sessions (*n* = 11 sessions; *n* = 5 mice). The gray dashed line shows that the 50% point from the fit across all sessions occurs at 22 ± 1 cells targeted.

**Extended Data Fig. 1:**
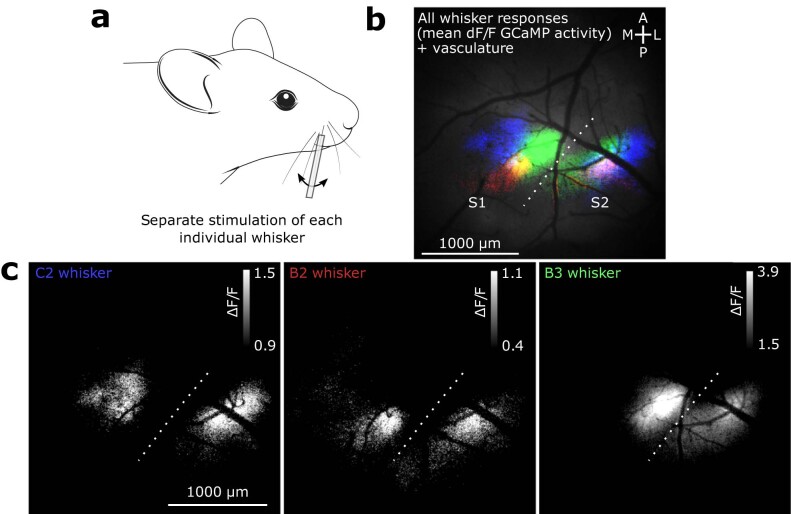
Mapping whisker S1 and S2 regions using widefield calcium imaging and whisker stimulation. **(a)** Single whiskers were deflected one at a time by threading them on to a capillary tube attached to a piezoelectric actuator. This figure was adapted with permission from Ethan Tyler and Lex Kravitz (10.5281/zenodo.3925901). **(b)** Each whisker was deflected multiple times and the results were averaged. The stimulus-triggered average responses from all whisker deflections is plotted on an image of the cerebral vasculature. **(c)** Individual stimulus-triggered average images from each whisker shows the topographical organisation of the barrel cortex in whisker S1 and the mirrored topography in whisker S2. Panels b) and c) show data from a representative single recording session.

**Extended Data Fig. 2:**
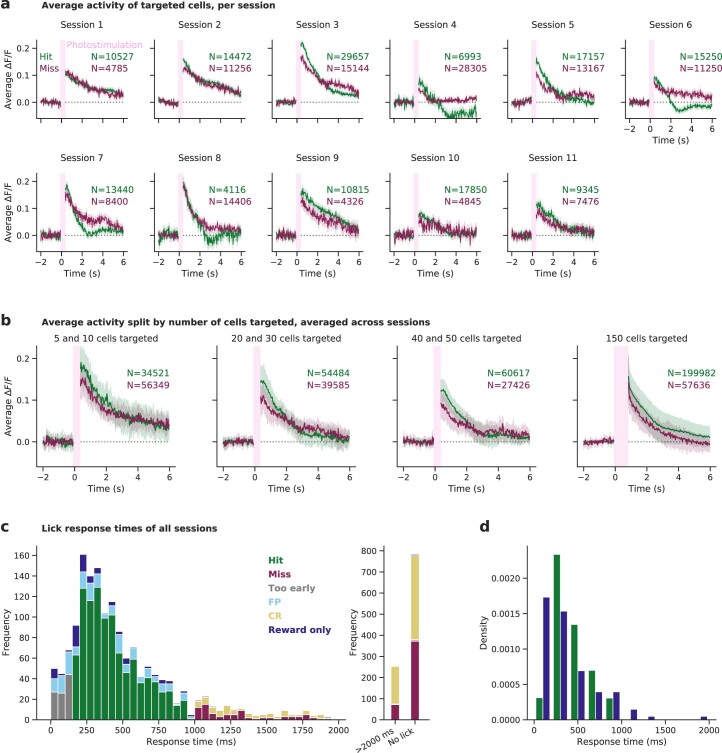
Activation of targeted cells by photostimulation. **(a)** The average ΔF/F activity of the cells targeted by photostimulation is shown (mean values +/− SEM) per recording session, for Hit and Miss trials separately. Targeted cells are activated similarly, with the notable exception of Hit-only inhibition at ~2 seconds post-stimulus in 3 recording sessions (of the same animal), indicating that the photostimulation-induced activation is largely independent of behavioural outcome. All trials with 5 to 50 cells targeted were used; trials where 150 cells were targeted were excluded because almost all of these trials resulted in a Hit outcome, biasing the averages. **(b)** The same data as panel a) is shown, but now averaged across animals and split by number of cells targeted (mean values +/− SEM). **(c)** Stacked histogram of the animals’ response times (defined by the time of the first lick) for each trial type. All trials with a response time within 2000 ms of all recording sessions are shown in the left panel, while trials with a response time greater than 2000 ms and trials where no lick occurred are grouped in the right panel. **(d)** Density histograms of response times of hit and reward only trials. Same data as in panel a), but normalised per trial type and binned per 200 ms. Medians (248 ms for reward only trials and 383 ms for hit trials) are significantly different (p = 0.01, (two-sided) Mood’s median test).

We developed a preparation that allowed us to record activity in two brain regions (S1 and S2) simultaneously while holographically photostimulating S1 (Fig. 1a). To achieve this, we expressed the genetically encoded calcium indicator GCaMP6s^33^ and the somatically targeted, red-shifted opsin C1V1-Kv2.1 (refs. ^21,34^) in layer 2/3 across both S1 and S2. We localized S1 and S2 by performing wide-field calcium imaging during deflection of individual whiskers (Fig. 1b and Extended Data Fig. 1). A field of view was selected that spanned the cortical representations of multiple whiskers across the a, b and c rows in both S1 and S2. Mice were head fixed, and we used a 2P microscope, based on previous designs^28^, adapted to perform 2P calcium imaging of neural activity across a large field of view (1.35-mm diameter) while performing 2P photostimulation of S1 neurons. Before each experiment, we photostimulated all opsin-expressing neurons in groups of 20 to find cells responsive to photostimulation, with neurons within a 350-μm diameter targeted simultaneously (Fig. 1c). Targeted neurons, defined as cells within 15 µm of the center of a photostimulation beam, elicited significant excitatory responses, whereas nearby S1 non-target neurons elicited significant inhibitory responses on average (Fig. 1d and Extended Data Fig. 2a,b; Wilcoxon signed-rank test, *P* < 0.05, Bonferroni corrected).

We trained mice by operant conditioning to associate direct photostimulation of neurons in S1 with water reward. After initial training, mice reported photostimulation by licking a spout (Fig. 1e). Crucially, to prevent a guessing strategy and/or random licking, mice had to withhold licking to initiate a trial after a variable inter-trial interval. Successful report of stimulation on ‘go’ trials was scored as a ‘hit’, and the mouse was rewarded; trials in which the animal failed to report the stimulation through licking were scored as a ‘miss’. We also randomly interleaved ‘catch’ trials in which no stimulus was delivered to analyze the detection performance relative to chance. Catch trials in which the animal licked were ‘false-positive’ trials, whereas trials when the animal appropriately refrained from licking were ‘correct-rejection’ trials (Fig. 1e). Between five and 150 photoresponsive S1 neurons were targeted on a single go trial. The number and identity of cells targeted were varied randomly on each trial to generate a psychometric curve (Fig. 1f), which shows that stimulating a greater number of neurons increased licking behavior accuracy. The 50% point of the psychometric curve fit across all sessions is 22 ± 1 neurons (*n* = 11 sessions recorded from five mice, ±95% confidence interval), in accordance with previously published findings^24^. Thus, we developed a preparation in which activity that causally drives behavior can be recorded, with single-cell resolution, both locally and after propagation downstream.

### 2P photostimulation of S1 drives balanced activity that propagates to S2

**Fig. 2:**
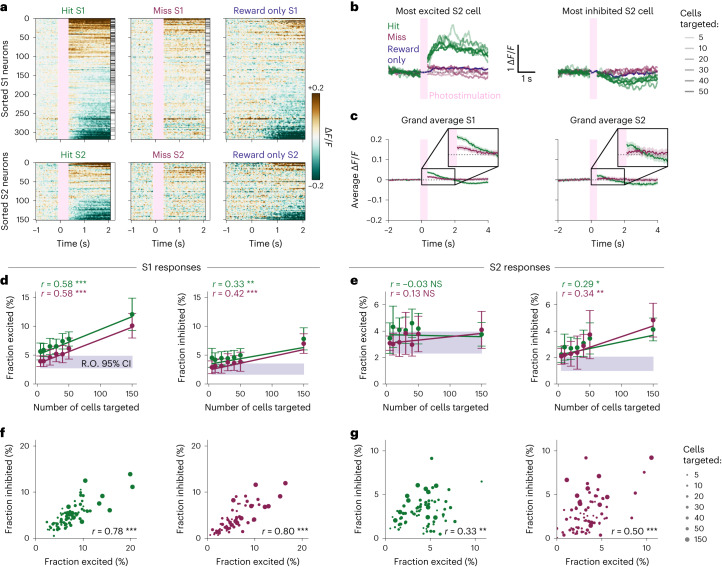
Activity driven by targeted 2P photostimulation is propagated from S1 to S2. **a**, Raster plots showing the trial-averaged response of different trial types (left: hit, middle: miss, right: reward only) to photostimulation (pink vertical bar: hit/miss) and/or reward (hit/reward only) of individual cells from a single session. (All trial types of all sessions are shown in Supplementary Figs. 1 and 2). Cells are sorted by the sum of their trial-averaged responses across all three trial types (Methods). Clear excitatory and inhibitory responses are elicited in S1 and S2 on hit trials that are not observed on miss trials or reward-only trials. The intensity of the grayscale bar on the right-hand side of the hit and miss rasters is proportional to the number of times each cell was directly targeted by the photostimulation beam, for hit and miss trials separately. Trials in which 150 cells were targeted were removed for display because their stimulation period is longer. Data are bound between −0.2 and +0.2 Δ*F*/*F* and blanked during the photostimulation (pink bar). **b**, Left: the Δ*F*/*F* activity traces of the most excited S2 cell (of the session of **a**) are shown, averaged across stimulus conditions. This S2 cell shows a large response on hit trials (green) but no response on miss trials (red) or reward-only trials (blue). The transparency of the line indicates the number of cells targeted in S1. Trials in which 150 cells were targeted were removed for display. Right: equivalent plot for the most inhibited S2 cell of **a**. **c**, The average population response to hit and miss trials across all sessions is shown (shaded areas show 95% confidence interval across trials of all sessions). Traces are averaged across cells, trials and sessions for a given trial type. Trials in which 150 cells were targeted were removed for display. The population responses of all other trial types are shown in Extended Data Fig. 3b,c. **d**, The neural response in S1 on hit and miss trials depends on the number of cells targeted in S1. Left: the fraction of excited cells (Methods) in S1 maps linearly to the number of cells targeted on both hit and miss trials. Right: the fraction of inhibited cells in S1 maps linearly to the number of cells targeted on both hit and miss trials. For hit and miss trials, data are presented as mean ± s.e.m. The shaded purple bar shows the 95% confidence interval across sessions of the fraction of excited or inhibited cells in S1 on reward-only trials. The linear fit was determined using weighted least squares, where the weights were the inverse variance of the trials that constituted a data point, and subsequently bound between their 25th and 75th percentiles to prevent extreme weight values. *P* values were computed using a two-sided *t*-test, where significance is indicated by ***P < 0.001, **P < 0.01, *P < 0.05 or NS (not significant). **e**, Equivalent panel for S1 responses. Left: There is no relationship between the fraction of excited cells in S2 and the number of cells targeted in S1 on hit trials or miss trials. The shaded purple bar shows the fraction of excited or inhibited cells in S2 on reward-only trials. Right: the fraction of inhibited cells in S2 maps linearly to the number of cells photostimulated in S1 on both hit and miss trials. **f**, The fraction of cells excited by photostimulation in S1 is highly correlated with the fraction of cells inhibited after photostimulation, both on hit trials (left) and on miss trials (right). The size of the circle indicates the number of cells photostimulated. The Pearson correlation coefficient is denoted by *r*, with significance indicated as before (two-sided *t*-test). **g**, The fraction of cells excited by photostimulation in S2 is correlated with the fraction of cells inhibited after photostimulation, both on hit trials (left) and on miss trials (right).

To understand how neural activity is propagated between brain regions to guide behavior, we compared neural responses during hit and miss trials, both in the directly stimulated region (S1) and the downstream region (S2). Qualitatively, hit trials elicit a diverse array of relatively strong excitatory and inhibitory responses both in S1 and S2, whereas both excitatory and inhibitory responses were less strong on miss trials (Fig. 2a and Supplementary Figs. 1 and 2). Excited and inhibited cells are also observed in ‘reward-only’ trials (Methods) in which reward was delivered in the absence of photostimulation; however, the neuronal responses are less pronounced compared to hit trials, indicating that the response to reported stimulation is not just explained by neural activity driven by reward (Fig. 2a and Supplementary Figs. 1 and 2).

**Extended Data Fig. 3:**
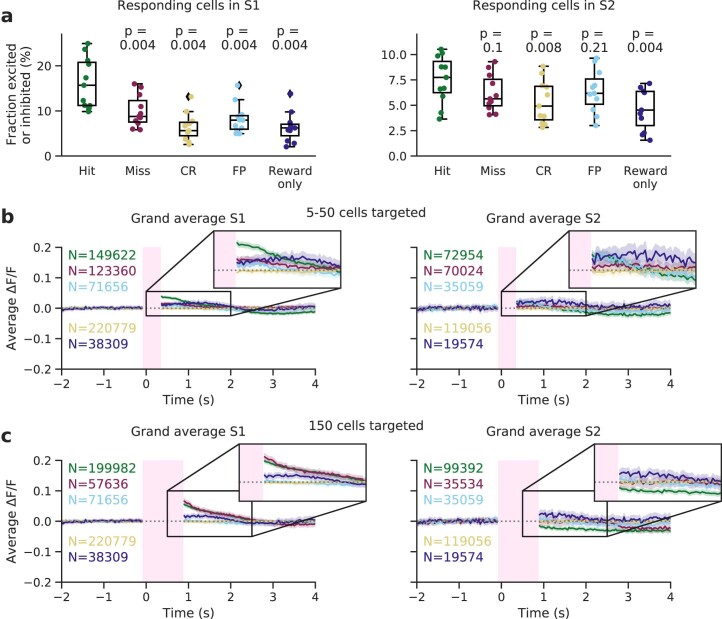
Fraction of cells responding and grand average traces of all trial types in S1 and S2. **(a)** The fraction of responsive cells (both excited and inhibited) on each trial type across all numbers of cells targeted. One-sided Wilcoxon signed-rank tests were used to test whether the fraction of responsive cells of hit trials was significantly greater than on other trial types (Bonferroni corrected for 8 tests). N = 11 independent recording experiments. Boxplots are defined by the median (centre), interquartile range (IQR) box, 1,5 IQR whiskers plus any outliers. **(b)** Average population responses of all trial types across all sessions. Traces are averaged across cells, trials and sessions for a given trial type. Trials in which 150 cells were targeted were removed for display (and shown in c). The time course of reward only trials is different from the time course of hit trials, hinting that the neural activity on hit trials constitutes more than motor preparation, movement, and reward related activity. This is further quantified in Figs. 2, 3. **(c)** As above but showing exclusively trials in which 150 cells were targeted.

We observed that hit trials elicit a significantly greater fraction of responsive cells than any other trial type in S1 (one-sided Wilcoxon signed-rank test, *P* < 0.05, Bonferroni corrected; Extended Data Fig. 3a). In S2, the fraction of responsive cells was more similar across trial types, suggesting that stimulus information is not encoded by this relatively simple metric (Extended Data Fig. 3a; one-sided Wilcoxon signed-rank test, Bonferroni corrected, *P* < 0.05, for hit, versus two out of four trial types). However, on a single-cell level, we found some individual neurons in S2 that are excited or inhibited in response to hit trials, but not miss or reward only, hinting that reported stimuli are propagated to single neurons in S2 (Fig. 2b).

Interestingly, however, averaging responses across cells, trials and sessions reveals that hit trials elicit only a modest increase in population activity from baseline in both S1 and S2 (peak post-stimulus Δ*F*/*F* = 0.038 ± 0.004 and 0.022 ± 0.006, respectively), followed by a prolonged period of inhibition, whereas, on miss trials, the population average response did not deviate from baseline in S2 (Fig. 2c).

The population responses of S1 and S2 suggest that excitatory stimulation is balanced by an inhibitory response (Fig. 2a). The relatively weak grand average response also supports that notion (Fig. 2c). Therefore, we analyzed the excited and inhibited cells separately. Indeed, the inhibited responses increased with the strength of optogenetic stimulation (number of cells targeted; Fig. 2d,e, right; correlation values are reported in the figure). In S1, but not in S2, the excitatory response also increased with stimulation strength (Fig. 2d,e, left). This may reflect the strong contribution of the targeted cells in S1.

To quantify this effect directly, we analyzed the excitation–inhibition ratio across all sessions. We found a clear correlation between the fraction of excited and inhibited cells for both hit and miss trials in S1 and S2 (Fig. 2f,g; correlation values are reported in the figure). This indicates that the excitation–inhibition ratio was maintained regardless of photostimulation strength or whether the stimulation was perceived. However, downstream from the stimulation, in S2, the correlation between excitation and inhibition was weaker than in S1 (excitation–inhibition correlation of S1 hit versus S2 hit, *P* = 1.2 × 10^−5^; S1 miss versus S2 miss, *P* = 0.001; two-tailed Steiger test; Fig. 2f,g). Taken together, we show that, as activity is propagated from its site of initiation in S1 to S2, the excitation–inhibition interplay persists but is less tight.

### S1 and S2 populations encode representations of perceived photostimulation

**Fig. 3:**
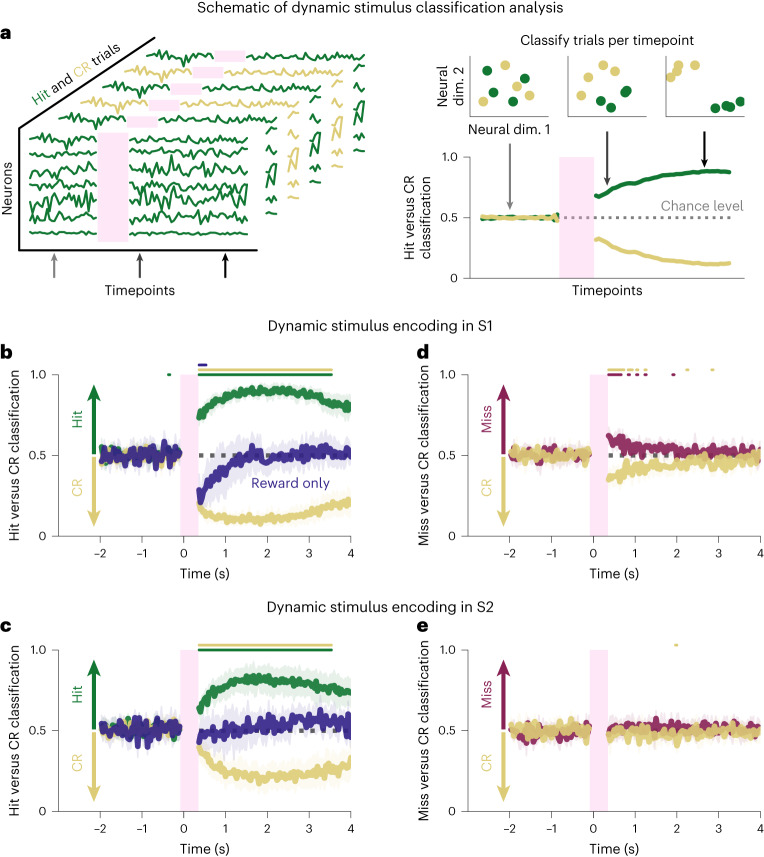
Perceived photostimuli elicit persistent activity in both S1 and S2 populations. **a**, The strength of trial type decoding in the neural population in S1 was dynamically quantified using logistic regression classifiers. Classifiers were trained on each timeframe individually, with activity of all cells in S1 or S2, and tested on held-out data. **b**, Classifiers were trained, for each timepoint, on S1 activity to classify hit trials from correct-rejection trials and then tested on held-out hit trials (green), correct-rejection trials (yellow) and reward-only trials (blue). Classifications are presented as mean ± s.e.m. across *n* = 11 sessions. Colored bars above the traces show timepoints at which classifier performance was significantly different from chance (two-sided Wilcoxon signed-rank test, *P* < 0.05, Bonferroni corrected; Methods). The classifiers were able to distinguish hit trials from correct-rejection trials with high accuracy for several seconds after photostimulation, implying that activity that arose from perceived stimulation persists in S1. Reward-only trials were not classified as hits, showing that the classifiers were not just trained to decode the neural signature of reward on hit trials. **c**, Classifiers were trained on S2 activity to distinguish hit trials from correct-rejection trials and then tested on hit trials (green), correct-rejection trials (yellow) and reward-only trials (blue). As above, the classifiers were able to decode hit trials from correct-rejection trials for several seconds after photostimulation, implying that activity that arose from perceived stimulation in S1 is propagated to S2 and persists for several seconds. Reward-only trials were not classified as hits, indicating that the model was not just detecting the neural signature of reward on hit trials. **d**, Classifiers were trained on S1 activity to classify miss trials from correct-rejection trials and then tested on miss trials (red) and correct-rejection trials (yellow). The classifiers were able to distinguish the two trial types for only for ~1 s after photostimulation. This implies that non-perceived stimuli do not generate persistent activity. **e**, Classifiers were trained on S2 activity to classify miss trials from correct-rejection trials and then tested on miss trials (red) and correct-rejection trials (yellow). The classifiers were not able to classify miss from correct-rejection trials, indicating that non-perceived stimuli were not robustly propagated from S1 to S2, likely because they were also not encoded in S1 (**d**). CR, correct-rejection; dim., dimension.

**Extended Data Fig. 4:**
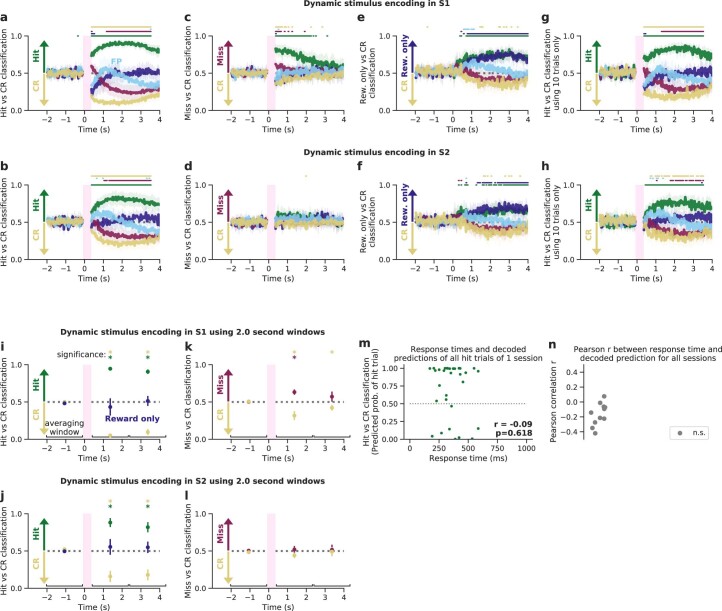
Dynamic decoders of all trial types. The strength of stimulus decoding of trial type in the neural population in S1 was dynamically quantified using logistic regression models. Models were trained on each time frame individually, with activity of all cells in S1 or S2, and tested on held-out data. **(a)** Models were trained, for each time point, on S1 activity to classify hit trials from correct rejection trials and then tested on held-out hit trials (green), correct rejection trials (yellow), reward only trials (dark blue), false positive trials (light blue) and miss trials (red). Classifications are presented as mean values +/− SEM across N = 11 sessions in panels (a)-(l). Coloured bars above the traces show time points at which classifier performance was significantly different from chance (two-sided Wilcoxon signed-rank tests, p < 0.05, Bonferroni corrected, see Methods). **(b)** As above, but trained on S2 activity. **(c)** Models were trained on S1 activity to classify miss trials from correct rejection trials and then tested on miss trials (red), correct rejection trials (yellow) reward only trials (dark blue), false positive trials (light blue) and hit trials (green). **(d)** As above, but trained on S2 activity. **(e)** Models were trained on S1 activity to classify reward only trials from correct rejection trials and then tested on reward only (dark blue), correct rejection trials (yellow), miss trials (red), false positive trials (light blue) and hit trials (green). **(f)** As above, but trained on S2 activity. **(g)** As (a) but the number of trials was restricted to 10 for each type, matching the total number of reward only trials recorded. **(h)** As (b) but the number of trials was restricted to 10 for each type, matching the total number of reward only trials recorded. **(i-l)** Equivalent to Fig. 3, but now using 2-second windows to train the classifiers. Three windows were used (−2.0 s up to and including −0.1 s, 0.4 s u/i 2.3 s, 2.4 s u/i 4.3 s), shown at the bottom of each panel. For each window, neural activity was averaged across time, per neuron per trial. Classifiers were then trained and evaluated as before and described in Methods. Asterisks indicate significant differences with chance level accuracy 0.5 (two-sided Wilcoxon signed-rank test, bonferroni-corrected p value < 0.05). As in panels (a)-(h), classifications are presented as mean values +/− SEM across N = 11 sessions in panels. **(m)** Comparison between lick response time and predicted outcome for all hit trials in S2. We considered the first decoder post-stimulus (at t = 367 ms), and compared for each trial the predicted outcome evaluated on withheld test data to the response time. This panel shows one example session (same session as Fig. 2a), and its Pearson correlation value r and associated (two-sided) p value. **(n)** Pearson correlation values r between response time and decoded S2 predictions of hit trials of all 11 sessions are shown. A Bonferroni multiple-comparison correction of N = 11 (sessions) was used.

**Extended Data Fig. 5:**
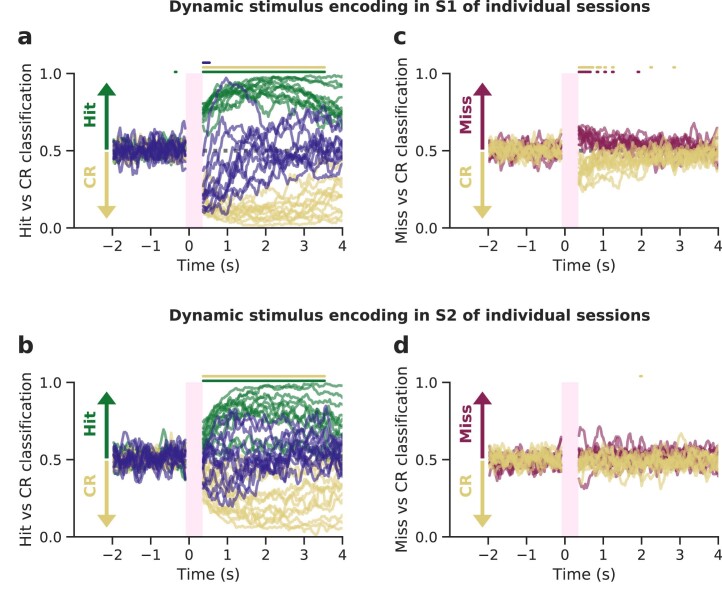
Dynamic decoders of individual sessions. **(a–d)** As Fig. 3, but displaying classifier performance for each individual session. Traces were smoothed using a running mean of 5 time points for visual clarity.

Individual cells displayed a diverse array of responses, and balanced population responses masked the magnitude and timecourse of propagated activity (Fig. 2). To gain insight into what task-related information is contained in these complex neural responses, we assessed propagation of stimulus-relevant activity from S1 to S2 and its relationship to behavior. We trained classifiers to dynamically decode the trial type of individual trials using the activity of all cells in S1 or S2 separately (Fig. 3a). First, we trained classifiers that distinguish hit trials from correct-rejection trials to detect the neural signature of both the stimulus and the activity resulting in its perception and report. Trained independently on each timeframe, the classifiers performed significantly above chance for more than 3 s after stimulation, both in S1 and S2 (two-sided Wilcoxon signed-rank test, *P* < 0.05, Bonferroni corrected for number of tested timepoints; Fig. 3b,c and Extended Data Figs. 4a,b and 5a,b). This shows that neural activity underpinning perceived stimulation persisted both locally in S1 and downstream in S2 for several seconds.

However, the classifiers could just have decoded the signatures of reward or the motor command required for licking, which are present on hit trials but not correct-rejection trials, instead of decoding stimulus perception. To disambiguate this, we tested the classifiers (that had been trained to distinguish hit versus correct-rejection trials) on reward-only trials (that is, trials in which reward was delivered in the absence of photostimulation). Performance on reward-only trials was either below or at chance level in S1 and remained at chance throughout the trial in S2, indicating that neural signatures of motor commands and licking were not the driving features of the classifier (Fig. 3b,c, blue lines, and Extended Data Fig. 4a,b; *P* > 0.05, Bonferroni corrected).

To evaluate the neural response to reward, we trained classifiers to distinguish reward-only from correct-rejection trials; for these classifiers, hit trials were evaluated at equal accuracy to reward-only trials (Extended Data Fig. 4e–h, dark blue and green traces). This indicates that the neural response related to reward is similar for both trial types and that neural signatures of perceived stimulation are present on hit trials, both in S1 and S2.

Next, we asked whether neural activity from unperceived photostimulation was reliably propagated from S1 to S2 by training models to classify miss and correct-rejection trials (Fig. 3d,e and Extended Data Figs. 3c,d and 5c,d). We found that, in S1, these classifiers decoded miss trials slightly above chance immediately after stimulation (*P* < 0.05, Bonferroni corrected); however, performance rapidly decayed back to chance level after 1 s. Conversely, in S2, the classifiers were not able to decode miss trials, indicating that stimulus information that is not perceived does not propagate to S2.

Taken together, these results show that only perceived stimulus representations are reliably propagated out of the local brain region in which they originate to a downstream brain area. Furthermore, the representations of stimuli that are propagated and drive perception persist both locally and downstream for several seconds after the injection of activity.

### Pre-stimulus S1 activity predicts trial outcome

**Fig. 4:**
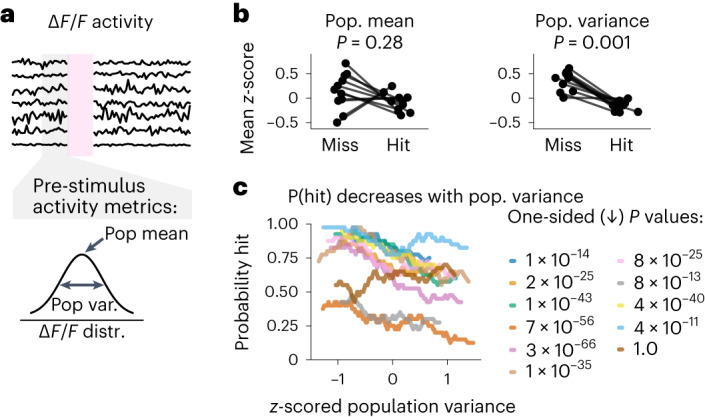
Pre-stimulus population in S1 predicts the upcoming trial outcome. **a**, Illustration of neural activity throughout a trial. Only the activity in the 0.5 s before the stimulus on a given trial is included in subsequent panels. First, we considered two metrics of pre-stimulus neural activity: the population mean and the population variance. **b**, Comparison of population metrics of pre-stimulus S1 activity before hit trials and before miss trials. Left: no evidence that mean population activity pre-stimulus predicts the upcoming trial outcome. Right: population variance is significantly higher before miss trials than before hit trials. Pre-stimulus population metrics in S1 were computed trial-wise and *z*-scored across trials within a session before being split into hit and miss trials and averaged across a session. *P* values were tested for a difference in session-wise population metrics between hit and miss trials (two-sided Wilcoxon signed-rank test). **c**, The probability of detecting the photostimulation decreased linearly with increasing pre-stimulus population variance in S1 for 10 of 11 sessions (one-sided *t*-test, Bonferroni corrected). Trials within a session were binned by their *z*-scored population variance, and this was correlated to the probability of a hit trial within that bin. distr., distribution; pop., population; var., variance.

**Extended Data Fig. 6:**
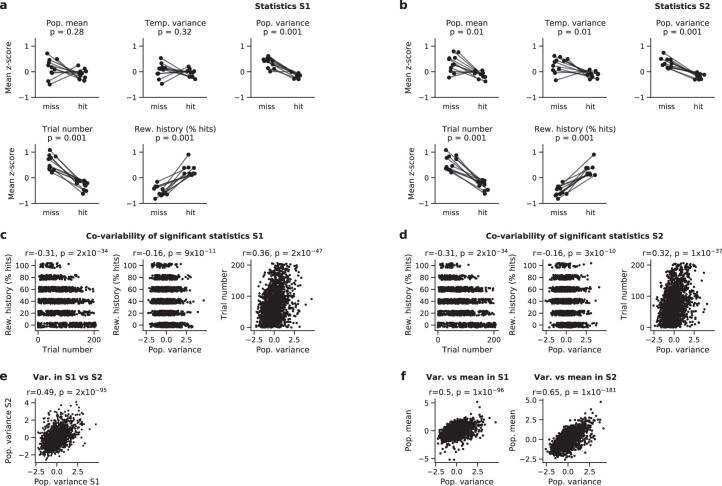
Further population metrics of pre-stimulus activity. Comparison of population metrics of pre-stimulus in S1 **(a)** and S2 **(b)** activity prior to hit trials and prior to miss trials. The first two panels show the two metrics of Fig. 4b. Next, two other significant task variables are shown; the trial number (an integer between 0 and the total number of trials in a session, indicating the number of trials previously undertaken by the animal in a given session) and the reward history (defined as % hit trials in the 5 preceding photostimulated trials). All variables were z-scored for clarity, and significance was assessed using two-sided Wilcoxon signed-rank tests. **(c, d)** The co-variability of the significant statistics of panels a and b was assessed using Pearson correlation (two-sided t-test). In sum, as recording sessions progress, reward history declines, and population variance is correlated to this trend. **(e)** Population variance is significantly correlated between S1 and S2 on a single-trial level (two-sided t-test). **(f)** Population variance is significantly correlated to the population mean on a trial-by-trial basis in both S1 and S2 (two-sided t-test). Population mean and variance were z-scored in all panels to allow comparison between different recording sessions. Trial number and reward history were z-scored in panels a-b only.

**Extended Data Fig. 7:**
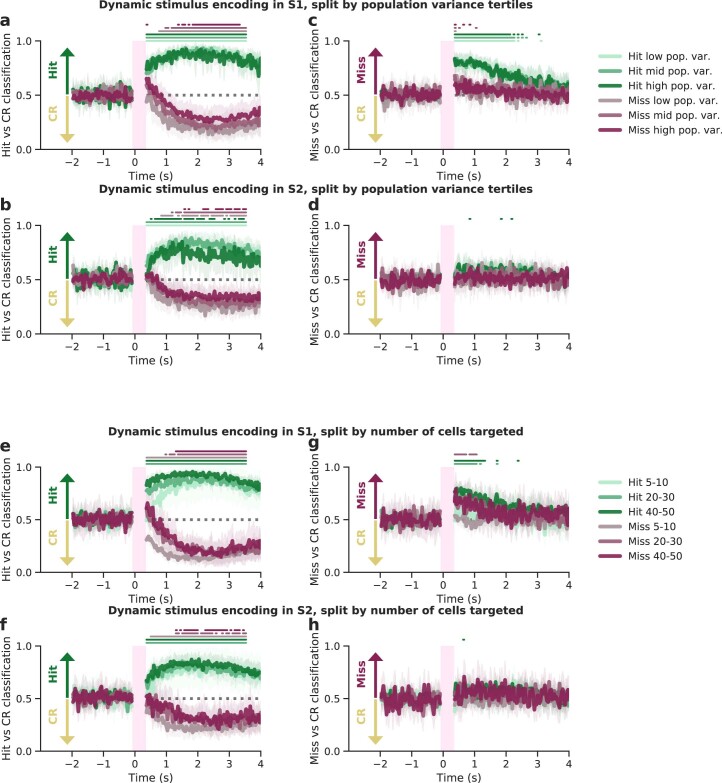
Dynamic decoding results split by population variance. **(a–d)** The decoders of Fig. 3 are shown, but additionally split by population variance. In other words, this figure shows the same data, but with an additional condition to separate trials by. Each recording session was split into 3 tertiles of equal size based on population variance (that is 3 bins were used), which were then averaged across sessions. Classifications are presented as mean values +/− SEM across N = 11 sessions. **(e-h)** The decoders of Fig. 3 are shown, but additionally split by number of cells targeted. Three ‘number of cells targeted’ conditions were used (instead of six) to increase data size, in particular for the rare scenarios (such as, for example, ‘Miss 40-50’). Two recorded sessions did not include data for one trial type/cells targeted combination, and were therefore omitted (for that combination only). Hence, classifications are presented as mean values +/− SEM across N = 9 sessions.

Although the previous analysis clearly showed that stimulation of S1 can be reliably propagated to S2 and reported behaviorally, it is unclear what intrinsic network conditions facilitate its detection. To identify the conditions that facilitate both the propagation to S2 and the behavioral report, we analyzed population activity in S1 immediately before stimulation (Fig. 4a) and asked whether the pre-stimulus population activity could predict whether the stimulus would be perceived (that is, whether the upcoming trial type would be a hit or a miss). To characterize population activity, we analyzed the distribution of instantaneous activity across neurons and computed the population mean and population variance as scalar measures. The mean population activity was not predictive of whether a trial will be a hit or miss (*P* = 0.28, Wilcoxon signed-rank test; Fig. 4b, left). In contrast, the population variance across S1 neurons strongly predicted whether the upcoming stimulus would be a hit or a miss trial. The population variance in S1 was larger before miss trials than before hit trials (*P* = 0.001, Wilcoxon signed-rank test; Fig. 4b, right). We further observed that population variance was correlated with task variables, such as trial number and reward history (Extended Data Fig. 6), and that population variance in S1 was correlated with that of S2 (Extended Data Fig. 6e). This suggests that population variance in both S1 and S2 may be a global mechanism underpinning how arousal and/or motivational state are instantiated in neural circuitry. The relationship between population variance and behavioral performance was also evident on a trial-by-trial level, whereby increased population variance pre-stimulus in S1 is negatively correlated with the probability that the upcoming stimulus elicits a hit (Fig. 4c; one-sided regression, Bonferroni corrected, *P* < 0.001, on 10 of 11 sessions). Furthermore, even though the population variance influenced the probability of the stimulus being perceived (and, thus, propagated downstream) (Fig. 4c), the subsequent strength of stimulus encoding was not influenced by population variance (Extended Data Fig. 7a–d). This implies that the neural representation of perceived stimuli was independent of the preceding population variance and, therefore, generalized.

### Strong pre-stimulus recurrent interactions before miss trials

Measuring population mean and variance immediately pre-stimulus is the most direct way of quantifying how instantaneous activity impacts perception (Fig. 4). However, more timepoints are needed to quantify the covariance structure, which could link these basic measures of population activity to mechanistic theories of circuit function. Therefore, we concatenated the activity that preceded all miss trials and hit trials separately and assessed their differences (see Methods and Supplementary Fig. 3a–c for details). We explored two complementary analytical approaches to characterize the role of population activity in driving behavior.

**Fig. 5:**
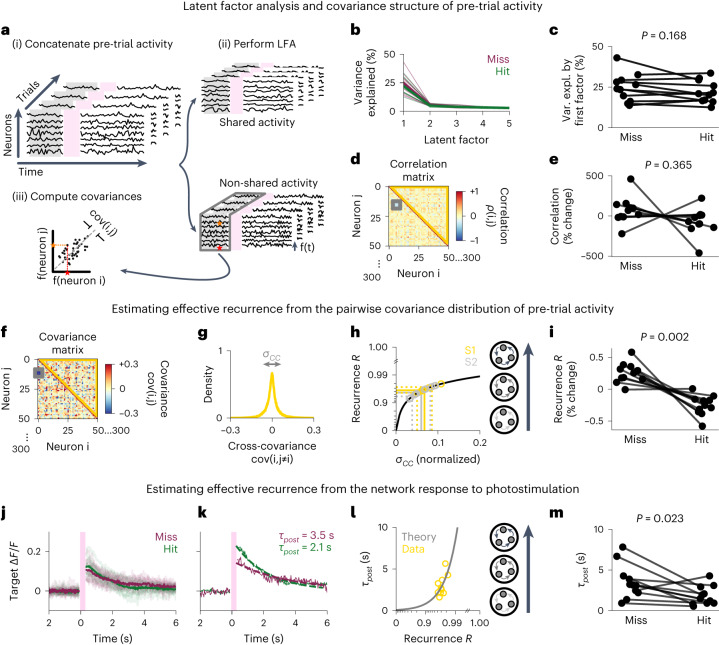
Miss trials are preceded by higher effective recurrence. **a**, To quantify effective recurrence *R* in a network, we first apply latent factor analysis to identify activity that is putatively driven by shared external input. We then subtract out this ‘shared’ activity and focus on the remaining ‘non-shared’ activity. **b**, Variance explained by each of the first five latent factors (miss: red, hit: green). **c**, The variance explained by the first factor (also called ‘across-neuron population-wise correlation’^35^; Supplementary Methods) is not significantly different between hit and miss trials (two-sided Wilcoxon signed-rank test, *P* = 0.168). **d**, For 6.5 s of non-shared pre-trial activity, the cross-neuron correlation matrix is calculated (orange and red stars show the process for a pair of example neurons; gray box highlights that of a single pair; yellow triangle marks the off-diagonal entries that are analyzed further). **e**, The mean off-diagonal cross-correlation is not significantly different before hit or miss trials (*P* = 0.37, two-sided Wilcoxon signed-rank test). **f**, Cross-covariance matrix of the non-shared activity (yellow triangle marks the off-diagonal entries used to compute recurrence). **g**, Histogram of the cross-covariance matrix; gray arrows indicate the variance of the distribution *σ*_*CC*_. **h**, The relationship between *σ*_*CC*_ and the effective recurrence *R* of the local network is known from theoretical derivations (Methods; plotted in black for a network of 50,000 neurons). Data for individual sessions are shown as gold and silver circles (S1 and S2, respectively); straight lines show the average across sessions; and dotted lines show the spread (mean ± s.d.). **i**, The effective recurrence *R* before stimulation is significantly different on hit and miss trials, suggesting that lower recurrence facilitates stimulus detection (*P* = 0.002, two-sided Wilcoxon signed-rank test). **j**, The average photostimulation response of the targeted cells on either hit or miss trials (excluding trials where 150 targets were stimulated, averaged across sessions in bold and averaged per session in shade). **k**, The ‘network response timescale’ *τ*_*post*_ was determined by fitting an exponential decay function per session. **l**, The inferred *τ*_*post*_ values (yellow circles) were better explained by the linear network theory (gray line, *r*^2^ = 0.44) than a simple linear regression (not shown, *r*^2^ = 0.38). **m**, The inferred network response timescale *τ*_*post*_ is significantly different on hit and miss trials (*P* = 0.023, two-sided Wilcoxon signed-rank test). expl., explained.

First, the strength and structure of inter-neuronal correlations can affect sensory-guided behavior^35,36^. Hence, we assessed whether the low-dimensional correlation structure of pre-stimulus population activity, measured as the variance explained by the first five latent factors, could predict the perceptual variability that we observed, but we found no evidence of this in our experiments (Fig. 5b,c; *P* = 0.168, two-sided Wilcoxon signed-rank test). We also tested whether the average ‘non-shared’ pairwise correlation of pre-stimulus activity was predictive, but, again, we found no evidence for this hypothesis (Fig. 5d,e; *P* = 0.365, two-sided Wilcoxon signed-rank test).

**Extended Data Fig. 8:**
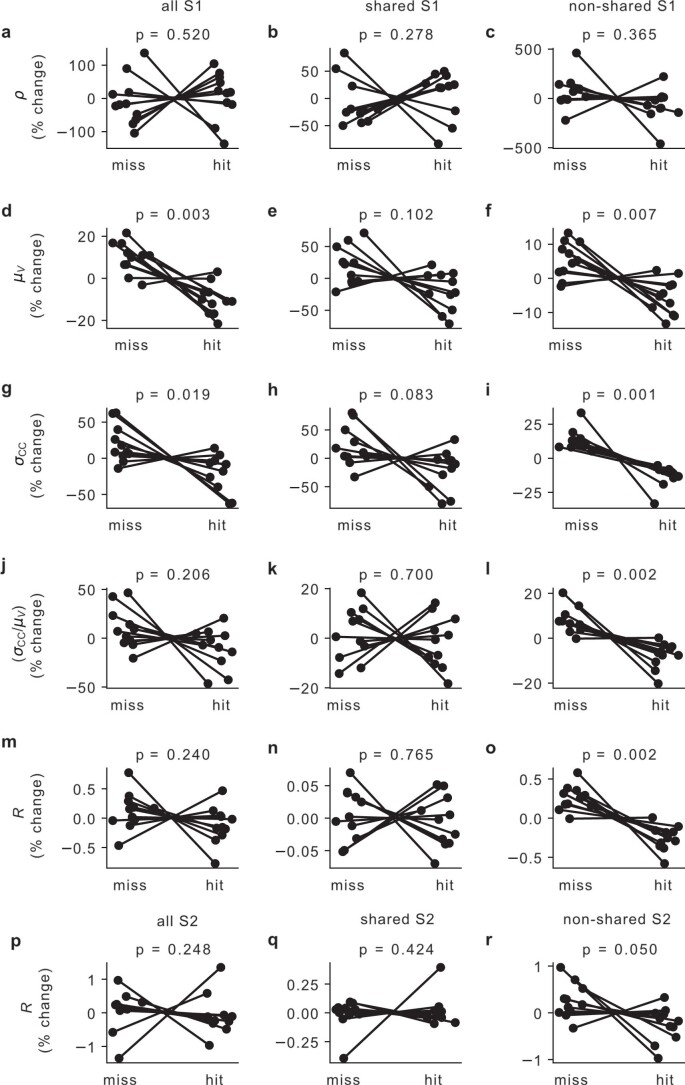
Recurrence analysis for all, shared and non-shared activity. The recurrence analysis results of Fig. 5 are shown in more detail. The first row **(a-c)** shows the mean off-diagonal correlation: for **(a)** all activity, **(b)** shared activity, and **(c)** non-shared activity. The second row **(d–f)** shows the mean on-diagonal covariance *μ*_*V*_. The third row **(g–i)** shows the standard deviation of the off-diagonal covariance *σ*_*CC*_. The fourth row **(j–l)** shows the ratio of the standard deviation of the off-diagonal covariance and the mean on-diagonal covariance *σ*_*CC*_/*μ*_*V*_. The fifth row **(m–o)** shows the change in recurrence R. The sixth row **(p–r)** also shows the change in the recurrence R, but estimated from neural activity in S2 instead of in S1. P values tested for a difference in session-wise population metrics between hit and miss trials (two-sided Wilcoxon signed-rank test without correction multiple comparison).

**Extended Data Fig. 9:**
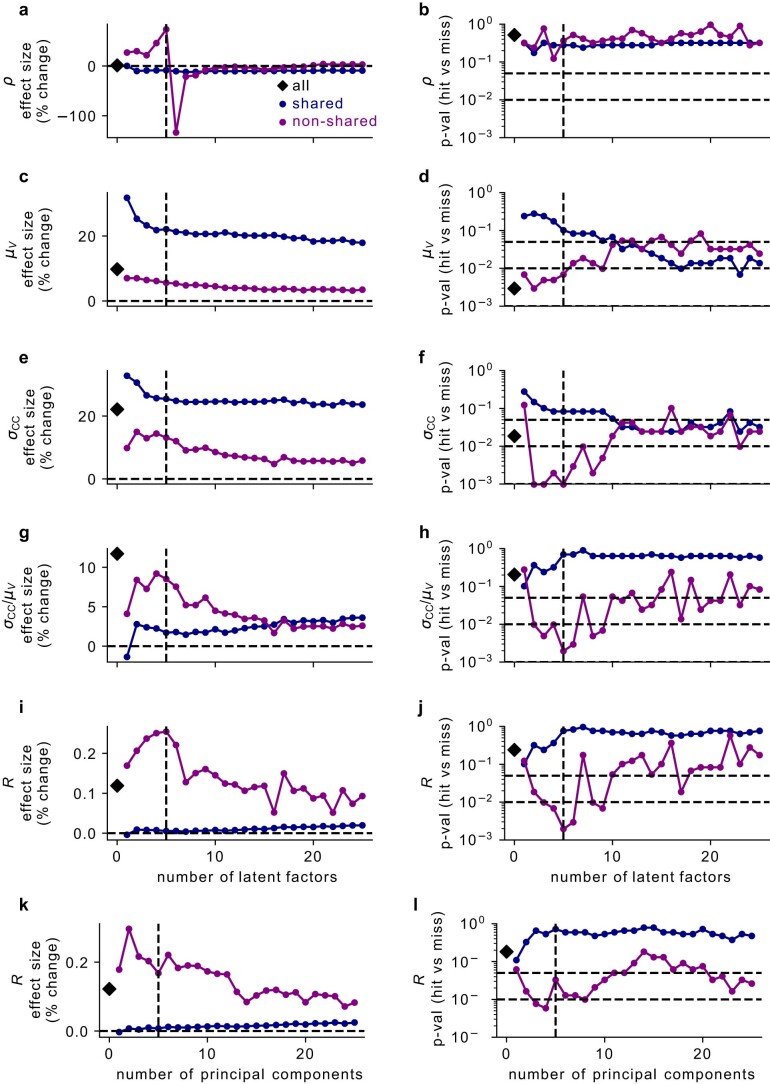
Recurrence analysis for different number of latent factors. The stability of the results shown in Fig. 5 with respect to the choice of the number of latent factors is explored (dashed vertical line denotes the 5 latent factors we used in Fig. 5). The first row **(a, b)** explores the hit-miss difference for the mean off-diagonal correlation; shown are **(a)** the effect size (dashed horizontal line denotes nil effect) and **(b)** the p-value of the observed effect (dashed horizontal lines denote p = 0.05, p = 0.01, and p = 0.001; two-sided Wilcoxon signed-rank test without correction multiple comparison), for all activity (black diamond), shared activity (purple crosses) and non-shared activity (pink crosses). The second row **(c, d)** explores the hit-miss difference for the mean on-diagonal covariance *μ*_*V*_. The third row **(e, f)** explores the hit-miss difference for the standard deviation of the off-diagonal covariance *σ*_*CC*_. The fourth row **(g, h)** explores the hit-miss difference for the ratio of the standard deviation of the off-diagonal covariance and the mean on-diagonal covariance *σ*_*CC*_/*μ*_*V*_. The fourth row **(i, j)** explores the hit-miss difference for the recurrence R. The fifth row **(k, l)** shows the same hit-miss difference for R, but uses Principal Component Analysis (PCA) instead of LFA to estimate shared activity.

Second, neural circuits are characterized by strong recurrence^37^, which is known to give rise to trial-to-trial variability^38^; moreover, the strength of recurrence can determine local amplification and encoding of external stimuli^39^. We inferred the strength of recurrence in the local network ($R$) from the non-shared covariance distribution as previously described^39^ (Fig. 5f–h and Supplementary Fig. 3), and we found that neural dynamics in both S1 and S2 are strongly recurrent ($R$ = 0.964 ± 0.008 in S1, $R$ = 0.957 ± 0.016 in S2; Fig. 5h), similar to other cortical areas in rodents^37,39^. Notably, we also observed that lower recurrence *R* before stimulation is correlated with higher detection probability (*P* = 0.002 in S1, Fig. 5i and Extended Data Figs. 8o and 9i,j; and *P* = 0.050 in S2, Extended Data Fig. 8r; two-sided Wilcoxon signed-rank tests). We next compared this network-wide, covariance-based recurrence metric to the network response timescale *τ*_*post*_, a measure of coupling strength inferred directly from the activity of the photostimulated targets^40^, and we found the same trend as for the recurrence *R* (Fig. 5j–m; *P* = 0.023, two-sided Wilcoxon signed-rank test). These data suggest that the previously described trial-by-trial differences in population variance might result from changes in the effective recurrence strength before stimulation, influencing the network’s sensitivity to direct stimulation.

### Stimulus strength and neural population variance determine probability of perception

Our previous analyses show that we have identified two separate conditions that predict the probability that a stimulus will be perceived: the strength of the stimulus (number of cells targeted; Fig. 1f) and the variance of the population activity (Fig. 4b,c). Next, we asked if these two predictors work in tandem—that is, whether stimulus perception depends on both the strength of the stimulus and the ongoing state of the population.

**Fig. 6:**
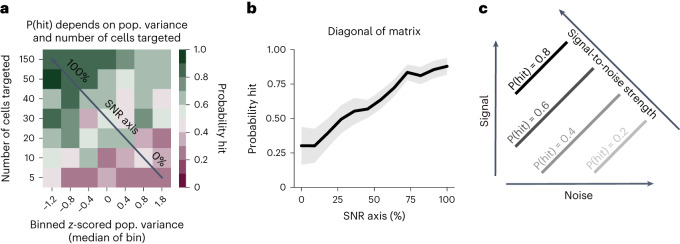
Stimulus strength and neural population variance underpin perception. **a**, The interaction between pre-stimulus population variance in S1 and the number of cells targeted by photostimulation defines the probability of a hit trial. Trials were binned by their *z*-scored population variance and by the number of cells targeted; the probability of a hit within each bin is plotted on a two-dimensional axis, pooled across all sessions. Increasing the number of cells targeted (that is, signal strength) and decreasing the pre-stimulus variance (that is, ‘noise’) generally yielded a greater probability of a hit trial. This is referred to as the SNR, as indicated by the diagonal black arrow. **b**, Maximizing the SNR of the stimulus resulted in the maximal probability of a hit trial. Data as in **a** but projected onto the SNR axis—as indicated in **a**—by averaging across all bins that project orthogonally onto each point on this axis. Data are presented as mean ± s.e.m. **c**, Schematic outlining the intuition for the SNR axis. Increasing the number of cells targeted on a given trial maximizes the signal of that stimulus. Noise is proportional to the population variance as there is more excitation and suppression from baseline in a population with high variance. The probability of hit is maximal when SNR is maximal, as the stimulus is more likely to be detected above ongoing activity.

We found that the probability that a stimulus elicits a hit can be expressed on a two-dimensional axis (Fig. 6a), where hit probability is highest when population variance is minimized and the number of cells targeted is maximized. More targeted cells were required to reliably drive hits, as pre-stimulus population variance increased. This phenomenon can be conceptualised by a signal-to-noise ratio (SNR) framework, in which activity injected into cells through photostimulation forms the signal, and the magnitude of background noise is measured by the population variance. The higher the SNR, the more likely the animal is to detect the signal above ongoing noise and respond, as quantified by collapsing the population variance and the number of cells targeted onto a single SNR axis (Fig. 6b; both population variance and the number of targeted cells contributed significantly to predicting trial outcome, both *P* < 10^−13^, logistic regression two-sided *t*-test; Methods). Furthermore, as pupil size changes have been associated with spontaneous activity fluctuations^10^, we measured the change in pupil size of three animals during the all-optical experiments, but we found no evidence that pre-stimulus pupil size consistently influenced trial outcome (Extended Data Fig. 9). In summary, our results show that the SNR of a stimulus determines how likely it is to be perceived and propagated downstream to drive behavior (Fig. 6c).

## Discussion

Critical to the brain’s ability to process stimuli is that activity is robustly propagated between functionally and anatomically distinct regions^9^. Although this question is often addressed in observational meso-scale studies of particularly the primate brain^41^, less is known about how the dynamics of neural activity are structured at the single-cell level to propagate activity between brain regions (but see refs. ^15–17^). By using an all-optical interrogation technique across multiple brain areas, we demonstrate, to our knowledge for the first time, that behaviorally salient photostimulation of S1 as a causal intervention elicits robust activation of S2. As expected, stronger stimulation elicits a stronger response in S2. Interestingly, we also found that pre-stimulus activity in S1 influences the detectability of the upcoming stimulus. This is consistent with an SNR framework in which the signal strength is the number of photostimulated cells and the noise is the pre-stimulus neural variability in S1. Thus, our results provide new insights into cortical function, as we show, to our knowledge for the first time, that the SNR of a stimulus, relative to network state, is critical for the propagation of the stimulus and its ability to drive action.

### Inhibition stabilization and generalization

One feature of cortical dynamics that may allow activity to reliably propagate is inhibition stabilization, in which inhibition tracks excitation to prevent unstable dynamics^42,43^. Indeed, we observed that excitation and inhibition covaried with stimulus strength in S1 and S2 and that both regions demonstrated correlated excitation and inhibition. Taken together, our results show that the population response of somatosensory cortex to excitatory photostimulation of small groups of neurons was stabilized by inhibition both in the excited region and in a downstream region. We observed that both the correlation between excitation and inhibition as well as the fraction of inhibited cells were lower in S2 than S1. This could explain why stimulus decodability was also slightly better in S1, as theoretical results suggest that strong inhibition enhances stimulus representations^44^. This stronger excitation–inhibition correlation in S1 may reflect stabilizing network dynamics in response to optogenetic perturbation, confirming predictions of how the activation of just tens of neurons can excite large cortical networks^45^.

Furthermore, we found that the population representation of the stimulus was generalized, as the neural response to perceived stimulation was similar across trials in both regions. We demonstrated this by showing that classifiers robustly decoded hit trials using cross-validation across trials, even though we varied the identity of targeted neurons on each trial. This variation of the stimulus requires sufficient consistency of the representation of the stimulus, implying that perceived neural activity remains generalized throughout its journey through the cortex. Conversely, on miss trials, the presence of the stimulus was decodable for only a brief period in S1 immediately after stimulation, and not at all in S2, showing that non-perceived stimulus information was not generalized across both brain regions. Stimulus generalization is a well-characterised phenomenon in psychology^46^, biological circuits^47,48^ and artificial neural networks^49,50^ and endows an agent with the ability to effectively interpret novel stimuli based on prior experience. This process has been shown to be enhanced if the stimulus is coupled to reward^48^, matching our results.

### Measures of neural variance

The SNR of a sensory neuron^51^ or population of neurons^52^ is often used to quantify the fidelity of the representation of a stimulus, whereby a higher SNR means that the sensory stimulus is more robustly represented in neural activity^15^. Indeed, one of the functions of the highly recurrent circuitry in sensory cortex is thought to be the amplification of relevant activity arising from feedforward inputs, to enhance SNR^15,53^. Despite the well-characterized importance of high SNR in the local representation of sensory stimuli, it is unclear how the SNR of a stimulus relates to its likelihood to propagate downstream. In this study, we generated a signal with direct cortical activation and quantified noise using the population variance metric. We found that the probability that a photostimulus was propagated and perceived was determined by the SNR of the stimulus.

Other metrics of pre-stimulus neural variability, such as single-cell temporal variance^10^, synchrony^54^ and neural oscillation power^15,55^, have all previously been linked to task performance. Although differences in recording techniques preclude direct comparisons, in particular, lower variance of single-cell membrane potentials was also found to enhance stimulus detection performance^10^, possibly providing a link between single-cell and population variability. Population variance, thus, captures the population-wide neural variability, which can arise from recurrent neural activity^39^.

We quantified the recurrence *R* to test theories^53^ about its impact on signal propagation. We found that the S1 network operates in a strongly recurrent dynamical regime, similar to other cortical areas in rodents^37,39^, monkeys^37,56^ and humans^57^, in which a small change in *R* can have a large effect. Indeed, we also found that slight variations around this operating point before stimulation reflect changes in detection performance. Specifically, we found that higher recurrence was associated with a decrease in stimulus detection. This is consistent with the SNR framework outlined above, whereby greater noise (variability) results in diminished perception, and with previous results that show that recurrence can be tuned to meet task requirements^56,57^. Our study adds to this body of literature with results obtained in a different model system (mouse) and different task (detection of direct cortical stimulation), further supporting the theory that differences in recurrence reflect changes in perceptual sensitivity.

### Causality and feedback connections

We suggest that the photostimulus is causal in driving both behavior and observed neural responses. Photostimulation was designed such that it causally drove behavior as the task required the mouse to lick after photostimulation to receive a reward. Our data confirm that, indeed, the probability of licking immediately after (sufficiently strong) stimulation was far greater than baseline. The causal paths that lead to the observed neural activity, in particular the transmission to S2, is less trivial because, in practice, causality is challenging to define^58^ or to quantify in the context of neural activity recordings^58^. One crucial criterion for causality generally, but particularly important in neuroscience, is that an effect should consistently follow a randomized intervention^58^. In our experiments, neural responses to perceived photostimulation in S2 were sufficiently consistent despite the random timing of their delivery such that we could classify them as specific to the hit condition. This resistance to randomization of the intervention critically underpins our argument for causality. Furthermore, our observed effects also satisfy some of the properties of observational causality (a weaker form of causality), namely strength (effect size), consistency (effect reliably follows stimulus), temporality (effect follows stimulus closely in time), dose–response relationship (greater stimulus leads to greater effect) and plausibility (reasonable mechanistic explanation)^58,59^. The strength, consistency, temporality and dose–response relationship of neural activity in S2 after S1 photostimulation are shown in Figs. 2, 3 and 6 and Extended Data Fig. 7e–h. The information transmission from stimulation to S2 neurons could be caused via many different paths. We do not have the experimental capacity to disentangle all potential paths driving S2 activity. These include, but are not limited to, direct, monosynaptic transmission from S1 to S2 (ref. ^31^), indirect paths via other brain regions^60^, brain state changes due to reward consumption^54^, reafference from the whiskers^61^ or movement^1^. Thus, although we can make statements about a causal relation, we cannot make any claim about the specific paths or their relative contributions.

Theoretical work has also highlighted the potential role of feedback connections in information processing^62,63^, and it is known that S2 also projects back to S1 directly and indirectly^31,64^. An interventional approach, for example^17^, could further interrogate the role of feedback in the S1–S2 circuit, to elucidate whether perception arises through a bi-directional flow of information.

### Technical limitations of the study

In this study, we focused on communication between two neighboring areas at the single-neuron level. We used state-of-the art recording and manipulation techniques (2P imaging and photostimulation), which require a tradeoff between spatiotemporal resolution and field-of-view size. For example, the ‘slow’ dynamics of GCaMP6s (the best-performing calcium sensor available at the time the experiments were performed) precluded us from reading out millisecond-level spike timings but allowed us to observe task-relevant activity of hundreds of neurons across a field of view spanning more than a square millimeter of cortex. Crucially, these slow dynamics also enabled us to read out the neural activity occurring immediately after trial onset despite the photostimulation artifact (but note that a combination of recent advances in sensor biotechnology and microscopy design could facilitate a higher temporal resolution^65–67^).

## Summary

In summary, we developed a new preparation to inject sparse, task-relevant activity into the cortex while recording its propagation downstream and resulting behavior. Using this, we elucidated how, at the level of neuronal networks, perception depends on the SNR of the stimulus relative to background activity. This reveals an organizing principle of the cortex that explains how inhibition-stabilized circuits can remain susceptible to behaviorally relevant stimuli.

## Methods

All experimental procedures involving animals were conducted in accordance with the UK Animals in Scientific Procedures Act (1986).

Male and female C57/BL6 and Tg(tetO-GCaMP6s)2Niell mice were used for all experiments. Mice were 4–12 weeks of age when surgery was performed. Mice were housed at room temperature (20–22 °C) on a standard light/dark cycle and humidity of ~40%.

### Surgical procedures

Animals were anaesthetised with isoflurane (5% for induction, 1.5% for maintenance) during all surgical procedures. A perioperative injection of 0.1 mg kg^−1^ buprenorphine (Vetergesic) and 5 mg kg^−1^ meloxicam (Metacam) was administered. Mice were prepared for chronic imaging experiments through a single surgery. Then, 2 mg kg^−1^ bupivacaine (Marcaine) was applied to the scalp before it was sterilized with chlorhexidine gluconate and isopropyl alcohol (ChloraPrep) before being removed bilaterally. The skull was cleaned with a bone scraper (Fine Science Tools) to remove the periosteum. An aluminium head plate with a 7-mm imaging well was bonded to the skull using dental cement (Super-Bond C&B, Sun Medical). A 3-mm circular craniotomy was drilled over the right somatosensory cortex, targeting the S1/S2 border (−1.9 mm posterior, +3.8 mm lateral) using a dental drill (NSK UK Ltd.). The skull within the craniotomy was soaked in saline before removal. Any blood was flushed with saline for more than 5 min, before a durotomy was performed. A single 1-μl viral injection was performed using a calibrated injection pipette beveled to a sharp point. Injections were performed at a rate of 100 nl min^−1^ at 300 μm below the pial surface and were controlled using a hydraulic micromanipulator (Narishige).

Pipettes were front loaded with either 1:10 GCaMP6s (AAV1-Syn.GCaMP6s.WPRE.SV40) diluted in C1V1-Kv2.1 (AAV9-CamKIIa-C1V1(t/t)-mScarlet-KV2.1) if injecting into C57/BL6 mice or C1V1-Kv2.1 alone if injecting into transgenic mice. After injection, a double-tiered cranial window composed of a 4-mm circular coverslip glued to a 3-mm circular coverslip was pressed into the craniotomy and sealed with cyanoacrylate (VetBond) and dental cement. Mice were recovered in a heated recovery chamber and kept under observation until behaving normally. Mice were subsequently monitored and their weight recorded for 7 d after surgery. Mice were allowed to recover for at least 21 d with ad libitum access to food and water before further procedures. This also allowed viral expression to ramp up before behavioral training was commenced.

### 2P imaging

2P imaging was performed using a resonant scanning microscope (2PPlus, Bruker Corporation) that raster scanned a femtosecond pulsed, dispersion-corrected laser beam (Vision-S, Coherent) across the sample at 30 Hz. A ×16/0.8 NA water immersion objective lens (Nikon) was used. GCaMP and mScarlet were imaged using a 920-nm and a 765-nm beam, respectively. Power on sample was controlled using a Pockels cell (Conoptics) and was kept at 50 mW for all experiments. A rectangular field of view (1,024 × 514 pixels, 1,397.4 × 701.4 μm) was used to image across two brain regions at 30 Hz. Imaging was controlled through PrairieView (Bruker Corporation). For 2P optogenetic procedures, see Supplementary Methods.

### Behavioral training

Mice were water restricted and given access to ~1 ml of water per day. Their weights were recorded, and ad libitum access to water or wet mash was provided if the animal’s weight dropped below 80% of the pre-restriction weight. For training, mice were head fixed using their head plate with their body supported in a 3D-printed polylactic acid tube. Mice became acclimatized to head fixation and relaxed in the tube after the first 1–2 sessions.

All behavioral training was controlled using pyControl hardware and software^68^ based around the micropython microcontroller. The pyControl framework acted as the master clock for behavior by writing the timing of behavioral input and output events to disk and triggering trials and stimuli based on behavioral events.

Mice reported photostimulation by licking a metallic lick spout placed ~5 mm from the tongue using a micromanipulator arm (Noga Engineering). The spout was electrically connected to the pyControl lickometer circuit (Open Ephys), which both recorded licking events and drove a solenoid valve (Lee Products) to deliver a ~2-μl water reward.

The general structure of the task and individual trials was consistent at all stages of training. Each trial was separated by a fixed 5-s inter-trial interval followed by a 4–6-s lick-withhold period, where the length of the lick-withhold period was drawn randomly from a uniform distribution spanning these times. This prevented mice from learning temporal structure in the task and eliminated the utility of a strategy based around random high-frequency licking. If the mouse licked during the lick-withhold period, the trial was restarted, and a new withhold length was drawn from the uniform distribution.

On trials where photostimulation was delivered, mice were rewarded if they licked to report perception of the stimulus. When no photostimulation was delivered, no punishment was administered for licking. No cues were made available that could signal the start of a trial.

The ‘response period’ during which the mouse’s licking response was recorded commenced immediately after the end of the lick-withhold period. This coincided with the onset of photostimulation in the case of go trials. The response period lasted for 1 s, and licks during this period alone were used to define the outcome of the trial. If the animal licked during the response period, this was scored as a ‘hit’; failure to lick on a go trial was scored as a ‘miss’. On catch trials, if the animal licked in the response period, the trial was scored as a ‘false positive’; trials where the animal did not lick in this period were scored as a ‘correct rejection’. A reward was delivered immediately after a correct lick on hit trials. This behavior can, thus, be considered a detection task where catch trials are used to report the animal’s baseline licking probability. Trial type was selected pseudorandomly ensuring no more than three consecutive trials of the same type. Mice were trained until they ignored 10 consecutive rewards or until 90 min had elapsed.

Behavioral performance was quantified using the d′ metric^69^, which quantifies the difference in response probability between hit and catch trials while controlling for baseline response rate. This allows for comparison of performance of mice with conservative licking strategies, that are less likely to lick on both go and catch trials, with mice that are more likely to lick on both trial types.

d′ is defined as

$$d{\prime} =z({hit}\,{rate})-z({false}\,{alarm}\,{rate})$$

where *z* is the Z-transform function

Sessions were discarded from all analyses if behavioral performance was poor, where d′ for trials on which 150 cells were targeted was less than 0.95 and/or d′ for trials on which 40 and 50 cells were targeted was less than 0.5.

### 2P behavioral training

Naive mice initially learned the association between photostimulation and reward through ‘one-photon’ wide-field stimulation with a 595-nm LED (Cree) (Supplementary Methods). After learning the one-photon stimulation task, mice were transitioned to the 2P version of the task, whereby mice responded to 2P photostimulation targeted to S1 only. Initially mice were trained on a task in which ~150 S1 neurons were photostimulated on every go trial in three groups of 50, with an inter-group interval of 5 ms (each group stimulated with 10 × 25-ms spirals; the entire 150-cell photostimulation takes 760 ms). Once mice registered a d′ > 1.5 across an entire session, they were transitioned to the main version of the task. This task consisted of three trial types selected pseudorandomly, with equal probability and with no more than three consecutive trials of the same type. On 1/3 of trials, 150 cells were targeted in groups of three; on 1/3 of trials, cells were targeted in a single group, with the number of targets drawn randomly from the set {5,10,20,30,40,50} with equal probability and with replacement; and the final 1/3 of trials were catch trials in which no photostimulation was performed. The 5–50-target photostimulation took 250 ms (10 × 25-ms spirals).

Before each session, photoresponsive cells were identified by performing 2P photostimulation spanning opsin-expressing areas of S1 (for example, Fig. 1c). This generated the coordinates of ~150 S1 neurons known to be responsive to stimulation. The subset of neurons to be targeted was selected randomly before each trial; cells in each simultaneously targeted subset were no more than 350 μm apart.

Before active behavior, 10 min of spontaneous imaging was performed without any photostimulation being delivered. During this time period, 10 rewards were delivered with an inter-reward interval of 10 s; this allowed us to assess the ‘reward-only’ neural response in somatosensory cortex. Active behavior followed spontaneous imaging, during which the mouse was rewarded only if it responded to a go trial. Neural activity was imaged throughout active behavior but was stopped every ~15 min to ensure that the objective lens was completely immersed in water and to monitor animal welfare. The field of view was manually corrected for drift throughout the session by moving the objective to realign the field of view to a marker cell.

White noise was played to the animal throughout the session to mask auditory cues signifying the onset of stimulation, and galvanometer mirrors were moved in an identical fashion on both go and catch trials. This ensured that the auditory cues generated were matched on both go and catch trials and also ensured that mice were responding to optical activation of S1 alone. Behavioral events were recorded through pyControl, and photostimulation was controlled by custom-written routines in Python and C.

### Imaging data analysis

Calcium imaging movies were processed using Suite2p^70^, and regions of interest corresponding to putative cell somata were manually selected. Suite2p also extracts a signal arising from the neuropil surrounding a cell body. To remove contamination of the signal arising from individual soma by the surrounding neuropil, we subtracted the neuropil signal from each cell body at each timepoint (*t*) according to the equation:

$$F(t)={F}_{{soma}}(t)-{F}_{{neuropil}}(t)\,x\,0.7$$

where:

*F* = neuropil subtracted fluorescence

*F*_*soma*_ = fluorescence from the cell’s soma

*F*_*neuropil*_ = fluorescence from the cell’s surrounding neuropil

0.7 = neuropil coefficient^33^

To ensure that cells with a bright baseline did not dominate the analysis, we computed Δ*F*/*F* for each cell using the equation

$$\varDelta F/F=(F-\underline{F})/\underline{F}$$

where:

$\underline{F}$ = the mean of $F$ across time through the entire session

Cells with very high Δ*F*/*F* values (max Δ*F*/*F* > 10), likely not arising from spikes, were discarded from further analysis.

Imaging data were split into individual trials, defined as 2 s preceding and 8 s following the onset of a trial. Trial onset was defined as the onset of photostimulation in the case of go trials, the onset of galvo spiraling in the case of catch trials or the delivery of reward in the case of reward-only trials. Frames occuring while the photostimulation laser was on were excluded due to artifactual crosstalk in the imaging channel (as well as two frames before and after stimulation to ensure that there was no contamination from the laser in neighboring timeframes). Due to the slow decay of the GCaMP6s calcium sensor, the magnitude (but not the precise timing) of neural activity obscured by photostimulation artifact was clearly apparent in the 500-ms post-stimulus period (Fig. 1d; see also Supplementary Fig. 4 for spike-to-fluorescence simulations of this effect). See Supplementary Methods for normalization and sorting procedures and definitions of targets, photostimulation responsiveness and excited/inhibited neurons.

### Normalization and sorting of post-stimulus neural activity

Post-stimulus neural activity was baselined relative to pre-stimulus activity to assess the relative change in activity after the photostimulation period and to compare this change across cells and trials. On each trial, for each individual cell, the average Δ*F*/*F* activity in the 2 s preceding the photostimulation was subtracted from the post-stimulus activity trace. This normalization procedure was applied to all analyses and visualizations of post-stimulus neural activity, except for Fig. 2d–f and Extended Data Fig. 3a where the difference between pre-stimulus and post-stimulus activity was assessed. Neurons were sorted for visual clarity only (in Fig. 2), using the sum of the post-stimulus Δ*F*/*F* activity on hit, miss and reward-only trials. This yields a sorting from strongly inhibited to strongly excited cells.

### Pre-stimulus population metrics

All pre-stimulus population metrics were computed across a 500-ms period immediately before photostimulation. All metrics were calculated on a trial-wise basis. The natural logarithm was taken of metrics that were fit better by a log-normal distribution as opposed to a normal distribution as assessed by Kullback–Leibler divergence (for clarity, this includes population variance).

Mean population activity was computed by first averaging Δ*F*/*F* activity across all pre-stimulus frames for each neuron to yield a vector containing a scalar value for each neuron defining its pre-stimulus activity. Next, firing rates were averaged across all neurons to give a single scalar value for each trial, defining the average population activity.

Population variance was computed in a similar fashion, first by averaging across all pre-stimulus frames (in the 500-ms window before stimulation) for each neuron. However, rather than taking the mean of the activity vector as above, the variance of the vector was used to generate a single scalar value for each trial.

### Logistic regression classifiers

The dynamic decoding classifiers of Fig. 3 used a logistic regression model with an L2 penalty term to the weights with a regularization strength of 0.001 (optimized by a parameter sweep with values 10^−7^ to 10^3^ with increments of factor 10). The Scikit-learn implementation of logistic regression was used^71^. Classification accuracy was computed per timepoint on a session-wise basis and then averaged across sessions, with shaded areas showing the 95% confidence interval of performance across sessions.

Each model was trained to classify the probability that a trial belonged to one of two different trial types. A 3:1 train:test split was employed, and model performance was assessed on held-out test trials only. Trials were subsampled if necessary to prevent biases (Supplementary Methods). Four-fold cross validation was used on each session, with a new model trained for each fold for each timepoint, and classification accuracy is reported as the average of the test data across folds, meaning that all (potentially subsampled) trials were in the held-out test set exactly once. A new model was trained from scratch for each imaging frame within a trial; hence, the training data consisted of a vector containing a single scalar Δ*F*/*F* value for each cell of all trials on a given frame.

See Supplementary Methods for more detail on the statistical tests used, classification of the reward-only trials, decoding time-averaged signals and analysis on the influence of response time, stimulus strength and population variance.

### Behavioral data analysis

As imaging was stopped intermittently, neural activity was not recorded for every trial performed by the animal; trials that were not imaged were excluded from all analysis. Hit trials in which the animal licked with an exceptionally short latency (<150 ms) are likely to have been driven by random licking rather than perception of the stimulus and were, thus, marked as ‘too-soon’ and not included in further analysis^24^.

For trial-wise analysis of neural activity, each trial’s time series was aligned to the onset of the (sham) photostimulation (time = 0 s) or to the onset of reward for the reward-only condition.

Psychometric curves were fit to behavioral data by computing the value of d′ separately for trials in which a given number of cells was targeted (Fig. 1f). This was achieved by comparing the hit rate for a given number of cells targeted to the false-positive rate across all catch trials. Data were fit using a logistic-function-adjusted lower bound at d′ = 0.

### Quantification of SNR effect

We performed a logistic regression analysis to validate that both stimulus strength (that is, the number of targeted cells) and the noise level (that is, pre-stimulus population variance) significantly contributed to predicting trial outcome (Fig. 6). Logistic regression was performed on the data of all hit and miss trials of all sessions (as in Fig. 6a,b). Both regressors significantly contributed to explaining trial outcome (both *P* values < 10^−13^, two-sided *t*-test on the logistic regression coefficients), with explained variance R^2^ of 8.4% (as calculated by McFadden’s pseudo-R^2^ for logistic regression). This was greater than the R^2^ obtained by regressing trial outcome to each of the two variables individually (R^2^ = 5.4% for number of targeted cells and R^2^ = 2.7% for population variance). Hence, trial outcome was best predicted using both regressors.

### Calculating the effective recurrence from the covariance matrix of non-shared activity

We infer the effective recurrence (*R*) from the covariance matrix of non-shared activity as described in ref. ^39^. In brief, the three main steps are (1) to disentangle the activity that is shared across all neurons from the non-shared activity that is individual to each neuron (latent factor analysis*)*. (2) From the non-shared activity, estimate the width of the cross-covariance distribution, which is an indicator of the dynamical state of the system, and the mean variance (two-step bias correction). (3) From the ratio of these two quantities, calculate the effective recurrence *R* directly as derived in ref. ^72^ (final estimate). To calculate the recurrence as precisely as possible but to avoid any bias from different trial numbers between conditions and sessions, we concatenate 6.5 s of pre-stimulation activity from 15 subsampled trials at a time and report the average of effective recurrence *R* across 1,000 subsamples for each session and condition.

### Calculating the network response timescale from photostimulated neurons

We calculated the network response decay time after photostimulation as a measure of persistent activity likely generated by local recurrence, adapted from ref. ^40^. To capture as much of the response as possible, we restricted our analysis to the subset of trials where photostimulation lasted 250 ms (that is, 5–50 cells targeted). For each trial, we selected only the targeted neurons and averaged their photostimulation response up to 5.5 s corrected for their average fluorescence in a 6.5-s pre-trial window. We then computed the average response across trials, subsampling 10 trials at a time 1,000 times to avoid any bias from different trial numbers between conditions and sessions. Last, we fit an exponential decay function with amplitude *A* and decay time *τ*_*post*_ to the averaged responses and, finally, reported the average of the decay time *τ* across 1,000 subsamples for each session and condition. To relate the network response timescale *τ*_*post*_ to the effective recurrence *R*, we used a simple network model^73^ that describes the firing rates of the targeted neurons **r**(*t*) as a linear function of the recurrent weights **W** and external input **h(*t*)**. After an eigenvector decomposition of **W** and identifying the largest eigenvalue as $R$, we found the decay timescale along its eigenvector *τ*_*post*_ to scale with $R$ as ${\tau }_{{post}}\sim \frac{1}{1-R}$.

### Statistics and reproducibility

We did not use statistical methods to determine sample size, and no randomization was used. Sample sizes were chosen based on previous studies^24^ that showed that statistical inferences can be drawn, in studies using 2P calcium imaging and 2P optogenetics, with *n* ≈ 10 mice. Data collection and analysis were not performed blinded to the conditions of the experiments. Unless otherwise stated, paired non-parametric tests were employed, and a *P* value of 0.05 was used as a threshold for significance throughout. Multiple comparison corrections were applied to significance tests using Bonferroni correction unless otherwise stated. Error bars show 95% confidence interval unless otherwise stated. We have made the code that (re)produces all analysis and visualization publicly available (see ‘Code availability’ section).

### Reporting summary

Further information on research design is available in the Nature Portfolio Reporting Summary linked to this article.

## Online content

Any methods, additional references, Nature Portfolio reporting summaries, source data, extended data, supplementary information, acknowledgements, peer review information; details of author contributions and competing interests; and statements of data and code availability are available at 10.1038/s41593-023-01413-5.

## Supplementary information

Supplementary Information Supplementary Figs. 1–4, Methods and References.

Reporting Summary

## Figures and Tables

**Extended Data Fig. 10:**
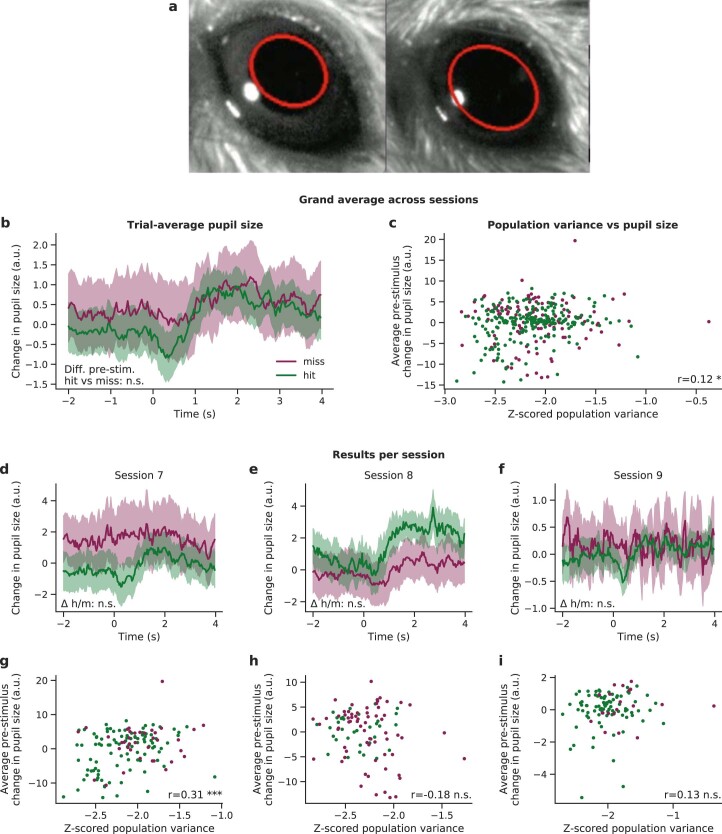
Pre-stimulus pupil size does not influence trial outcome. **(a)** Pupil size was measured for 3 sessions (sessions 7, 8 and 9, corresponding to Supplementary Fig. 2), see Methods. **(b)** Trial- and session-averaged pupil size dynamics for hit and miss trials, where shaded areas indicate the 95% confidence interval of the mean (across sessions and trials). The time-averaged pre-stimulus pupil size was not significantly different between hit and miss trials (two-sided t-test). **(c)** Population variance was very weakly correlated to pupil size across all sessions (both averaged across 500 ms pre-stimulus per trial) (two-sided Pearon correlation r = 0.12, p = 0.02). **(d–f)** Trial-averaged pupil size dynamics per session, where shaded areas indicate the 95% confidence interval of the mean (across trials). Pre-stimulus differences between hit and miss were not significant for any of the sessions (two-sided t-tests), although session 7 (panel d) almost reached the significance threshold (p = 0.053). **(g–i)** Population variance vs pupil size per session. Two-sided Pearson’ correlation was significant for only one of the sessions (session 7).

## Data Availability

All source data used for the data analysis and visualization are publicly available on a GIN data repository at 10.12751/g-node.h27xvl. This includes the 2P calcium imaging and optogenetics recordings of all experiments as well as the pupil size data.

## Code availability

Behavioral training used pyControl hardware and software as previously reported^68^. Photostimulation was controlled by custom-written code in Python and C, available from the authors upon reasonable request. Online imaging analysis used STAMovieMaker (https://github.com/llerussell/STAMovieMaker). Offline pre-processing imaging analysis was performed using Suite2p^70^.

All subsequent data analysis and visualization were performed in Python 3.7 using custom-written code, which we have made publicly available on GitHub at 10.5281/zenodo.8066289.

Furthermore, the recurrence and pupil imaging processing analysis can be found on GitHub at 10.5281/zenodo.8109935.
