# Supplementary material for: Propagation of activity through the cortical hierarchy and perception are determined by neural variability
Source: Nat Neurosci. 2023 Aug 28;26(9):1584–94. doi: 10.1038/s41593-023-01413-5 (PMC10471496; doi:10.1038/s41593-023-01413-5)
Supplement: Supplementary file 1 — Supplementary Figs. 1–4, Methods and References. [file 41593_2023_1413_MOESM1_ESM.pdf]

nature neuroscience

Article https://doi.org/10.1038/s41593-023-01413-5

Propagation of activity through the cortical hierarchy and perception are determined by neural variability

In the format provided by the authors and unedited

Supplementary Information: Perception and propagation of activity through the cortical hierarchy is determined by neural variability

James M. Rowland[1*], Thijs L. van der Plas[1*], Matthias Loidolt[1,2,#*], Robert M. Lees[1], Joshua Keeling[1], Jonas Dehning[2], Thomas Akam[3], Viola Priesemann[2,4], Adam M. Packer[1]

*These authors contributed equally to this work

[1]Department of Physiology, Anatomy, and Genetics, University of Oxford, Oxford, UK

[2]Max Planck Institute for Dynamics and Self-Organization, Göttingen, Germany.

[3]Department of Experimental Psychology, University of Oxford, Oxford, UK

[4]Institute for the Dynamics of Complex Systems, University of Göttingen, Göttingen, Germany.

[#]Present address: Laboratory for Molecular Cell Biology, University College London, London, UK

Supplementary Figures

Supplementary figures and figure legends follow this page.

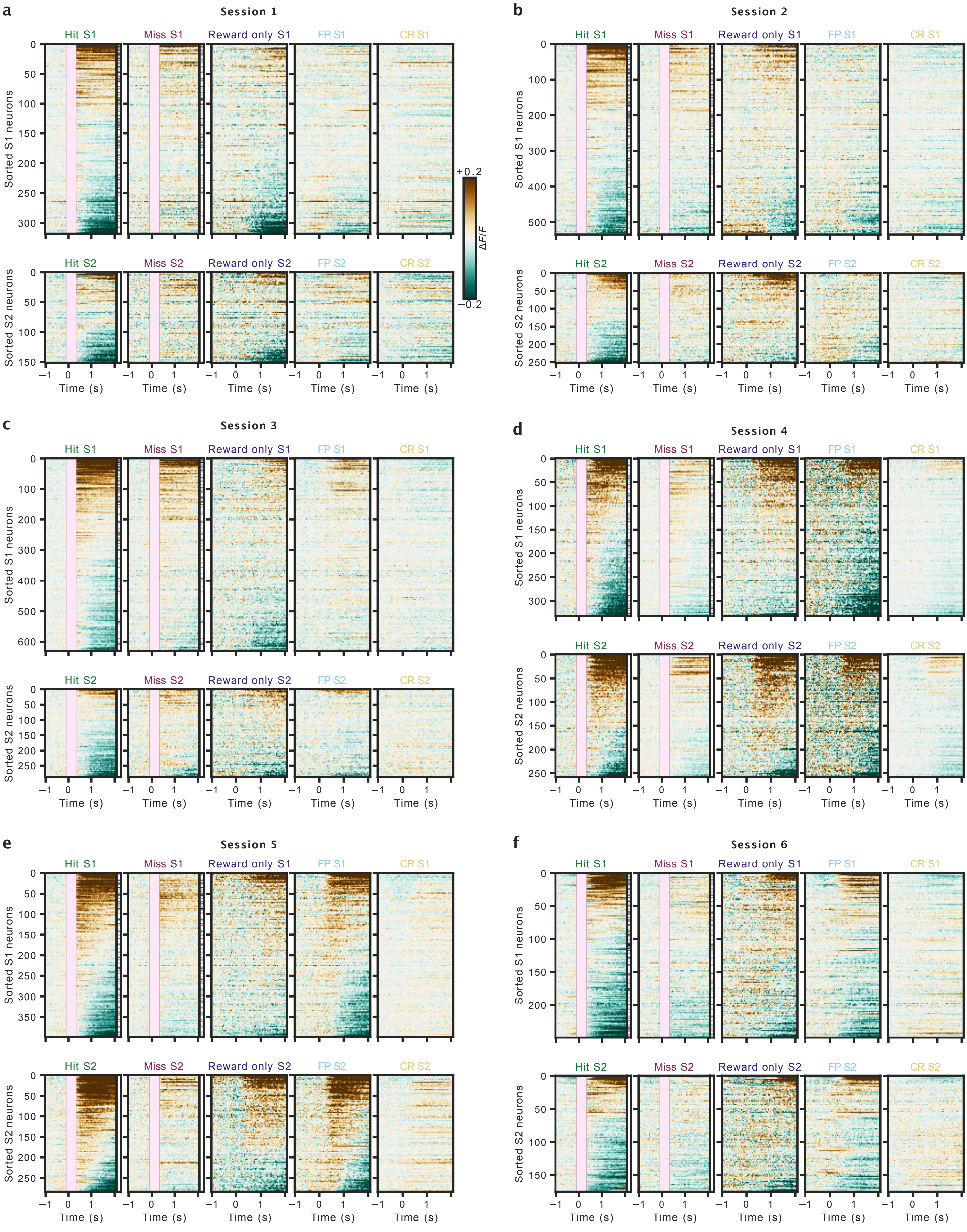

Supplementary Figure 1

Supplementary Figure 1: Trial-averaged raster plots of all sessions and trial types

(a-f) Raster plots showing the trial-averaged responses for all trial types to photostimulation (pink vertical bar, hit/miss) and/or reward (hit/reward only) and/or licks (hit/reward only/false positive) and none of these (correct rejection) of individual cells from the first 6 sessions.

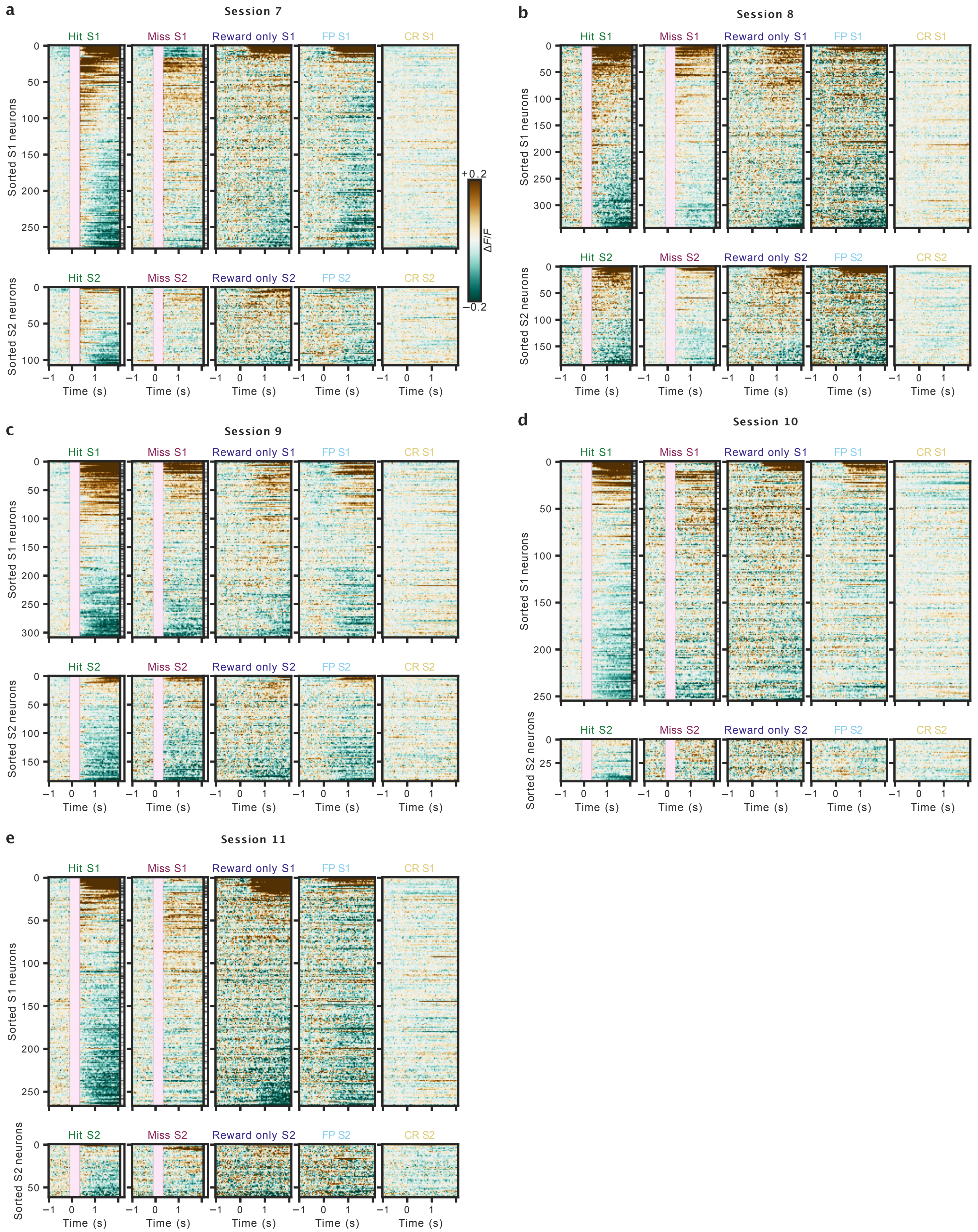

Supplementary Figure 2

Supplmentary Figure 2: Trial-averaged raster plots of all sessions and trial types (continued)

(a-e) As above for the remaining 5 sessions.

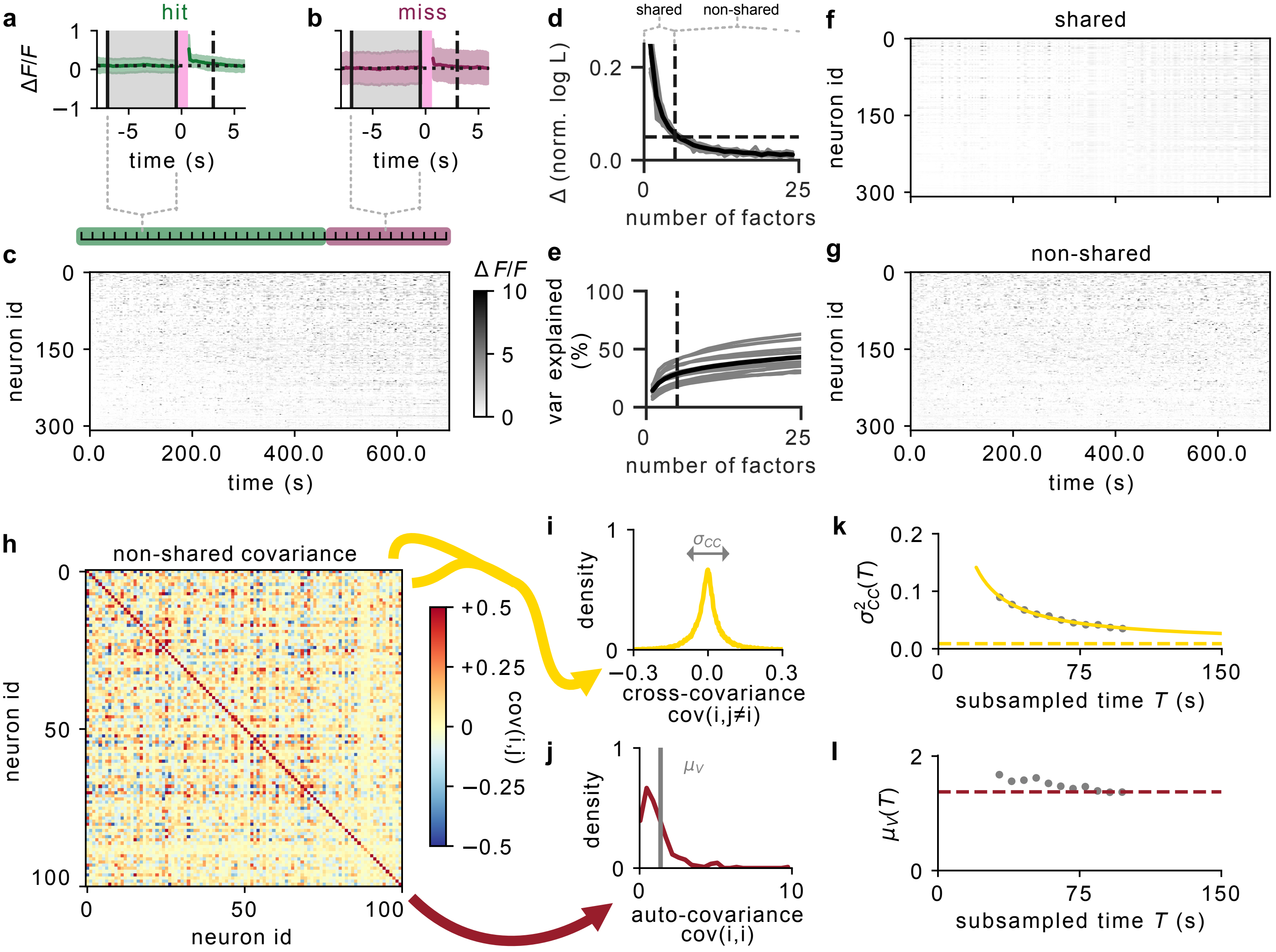

Supplementary Figure 3

Supplementary Figure 3: Inferring the effective recurrence from non-shared covariances

(a) Trial-averaged peri-stimulus average activity of S1 during hit trials in an example session (Mouse RL123, run 22, 87 hit trials). The green line and shaded region show the mean ΔF/F and variance across all S1 neurons and all hit trials. The two vertical solid black lines are the bounds of the (pre-stimulus) analysis period, which is coloured in grey. The single vertical dashed black line also denotes the start of the analysis period, but here referenced to the preceding stimulation (the earliest possible start, as the inter-trial-interval varies). The horizontal dotted black line denotes the mean population activity during the pre-hit period. The pink bar denotes the photostimulation period. **(b)** Trial-averaged peri-stimulus average activity of S1 during miss trials (red). **(c)** Pre-stimulation activity of S1 neurons concatenated across all 87 hit and 21 miss trials, trial structure shown at the top is for visualisation only and not to scale. **(d)** Increase in log-likelihood of the LFA fit (min-max normalized) as a function of the number of latent factors performed across all analysis windows preceding hit and miss trials. Grey lines show individual sessions, the black line shows the mean across sessions. The dashed horizontal line shows the 5% threshold that was used to determine the number of factors, the dashed vertical line shows the 5 factors we selected to calculate the shared activity, all other factors were discarded and the non-shared activity is used for further analysis. **(e)** Percentage of the variance explained by the LFA as a function of the number of latent factors. **(f)** Shared pre-stimulation activity of S1 neurons concatenated across all hit and miss trials, as calculated by projecting the first 5 latent factors back onto the neurons. Colour scale is the same as in **c**. **(g)** Non-shared pre-stimulation activity of S1 neurons concatenated across all hit and miss trials, as calculated by subtracting the shared activity. Colour scale is the same as in **c**. **(h)** Covariance matrix of non-shared activity in S1, i.e. the activity that is not common across most recorded neurons, that is, not explained by the main 5 latent factors.

Only 100 out of 309 neurons are shown. **(i)** Distribution of off-diagonal components $c_{ij, i \neq j}$ of the non-shared covariance matrix shown in (a). The grey arrow denotes the standard deviation σ_{CC} of the on-diagonal components. **(j)** Distribution of on-diagonal components c_{ii} of the non-shared covariance matrix shown in (a). The grey line denotes the mean μ_V of the on-diagonal components. From the ratio of σ_{CC} and μ_V, the effective recurrence R can be inferred. **(k)** Artificial sub-sampling procedure to obtain an estimate of the standard deviation σ_{CC} of the off-diagonal covariances that is not biased by the finite number of bins T recorded in a single session. Grey points show the average of $\widehat{\sigma_{CC}^2(T)}$ for 50 random samples of $T = [5 * 65, 6 * 65, \ldots, 15 * 65]$ bins chosen without replacement, yellow line shows the fit of the relation $\widehat{\sigma_{CC}^2(T)} = \frac{const.}{T} + \sigma_{CC}^2(\infty)$, and the dashed yellow line shows the asymptote $\sigma_{CC}^2(\infty)$ obtained from said fit. **(l)** The mean of the on-diagonal covariances is not significantly biased by the finite number of bins T, hence we use the value obtained for $T = 15 * 65$ bins (dashed red line).

Synthesized fluorescence response to single spikes

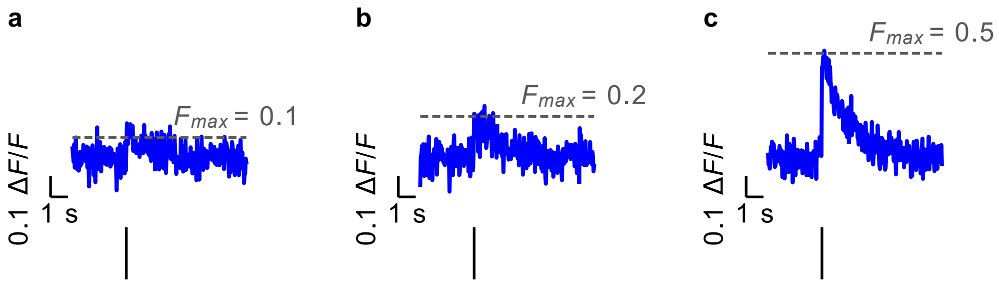

Synthesized fluorescence response to five 'hidden' spikes

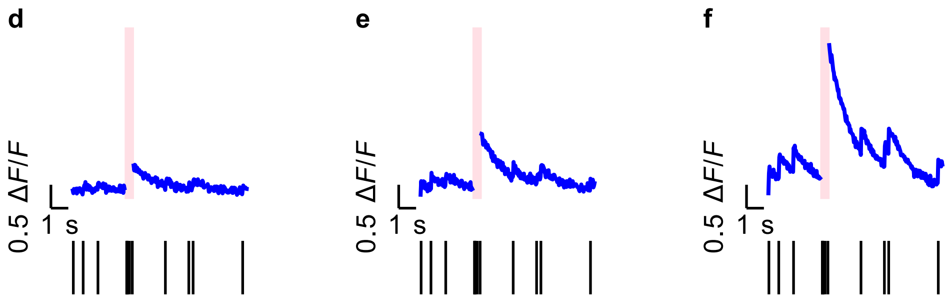

Supplementary Figure 4

Supplementary Figure 4: Early trial activity is reflected in calcium imaging traces despite the photostimulation artifact

(a-c) Synthesized fluorescence response to single spikes for three different simulation conditions, varying the fluorescence induced by a single spike (F_{max}) while keeping all other parameters equal. The black vertical bar denotes the timing of the spike, blue traces show the synthesized fluorescence response, horizontal dotted grey line shows the F_{max} parameter.

(d-f) Synthesized fluorescences response to five 'hidden' spikes occurring during the photostimulation window in the presence of a 1 Hz Poisson spiking background are shown, for the three different simulation conditions outlined above. Black vertical bars denote the timing of the spikes, blue traces show the synthesized fluorescence responses, pink vertical bar denotes the photostimulation window (500 ms long, 'hidden' spikes are inserted 110-150 ms after photostimulation onset).

Supplementary Methods

One-photon behavioural training

One-photon behavioural training was carried out in closed, light-proofed wooden boxes with the LED placed directly onto the cranial window. Current was supplied to the LED using pyControl and a custom designed analog driver board, allowing the power produced by the LED to be controlled programmatically. LED power was calibrated using a power meter (ThorLabs PM100D). A single trial's photostimulation consisted of 5 x 20 ms pulses over a period of 200 ms. LED stimulation was delivered on go trials only.

Initially, mice learned the association between optogenetic stimulation and reward through conditioning in which the stimulus was paired with reward regardless of the animal's response. Rewards that were delivered on miss trials are termed 'auto-rewards' and were delivered at the end of the response period. Mice rapidly learned to associate the optogenetic stimulus with reward and began licking before the auto-reward within 1-2 sessions. Mice were transitioned out of the auto-reward phase and into active training when they scored three consecutive hit trials. During active training (including subsequent two-photon behavioural training, see below), mice were not auto-rewarded aside from on a single trial if they registered 3 consecutive misses; this functioned to motivate poorly performing mice during a session. These auto-rewarded trials were excluded from all further analyses. On rare occasions (33 out of 1043 total hit trials), lick responses just before the end of the response window (928 - 985 ms interquartile range from photostimulus onset) were registered just outside the response window and therefore not rewarded. These were also excluded from all further analyses.

During the initial training phase and the first stage requiring active responses, LED power of 10 mW was used, with mice transitioned to progressively weaker LED powers once their performance was sufficiently high. Online performance was computed using running d', computed across a window of 10 trials. Once animals had reached a running d' of 2, the LED power was reduced in a stepwise fashion, and the running window was reset, until the lowest power (0.1 mw) was reached. Once animals had exceeded criterion at this power, they were considered to have completed the one-photon training paradigm.

Widefield imaging

Whisker stimulation during widefield calcium imaging was used to: target virus/tracer injections, determine the viability of a preparation for continued experimentation and find a suitable field of view for two-photon imaging encompassing S1 and S2 responses to a single whisker deflection. Using a camera, an LED and a dichroic/filter set, the whole cranial window was imaged using epifluorescence to measure calcium responses to whisker deflections (**Extended Data Fig. 1)**. Each one of four whiskers (B1-B3 and C2; one at a time) was threaded into a capillary and deflected for 10 trials of 1 second each with 10 second inter-trial intervals. Each stimulation was 10 Hz and of ~300 um in amplitude at ~500 um from the base of the follicle. Responses to whisker stimulation were assessed using ΔF/F stimulus-triggered averages based on a baseline of 1 second pre-stimulus. Two areas of response moved stereotypically anterior-posterior and medial-lateral according to the row and column of the whiskers stimulated, confirming whisker responses were being assessed (**Extended Data Fig. 1b, c**).

Two-photon optogenetic stimulation

Two-photon optogenetic stimulation was conducted using a pulsed fixed-wavelength fibre laser at either 1030 nm (Satsuma HP2, Amplitude Systèmes) or 1035 nm (Monaco, Coherent) at a repetition rate of 2 MHz. Multiple individual neurons were targeted for stimulation simultaneously by splitting the laser beam using a reflective spatial light modulator (SLM) (7.68 x 7.68 mm active area, 512 x 512 pixels, Boulder Nonlinear Systems). The active area of the SLM was overfilled and the polarisation optimised for maximal first order diffraction efficiency using a half-wave plate. The zero order diffraction beam was blocked using a well bored into an optical flat using a dental drill (NSK UK Ltd).

Phase masks were loaded onto the SLM using the Blink SDK (Meadowlark Optics). Phase masks were computed by applying the Gerchberg-Saxton algorithm[1] to the xy coordinates of the target cell bodies. A weighted form of this algorithm was used to ensure uniform power distribution across all cells as the first order diffraction efficiency of the SLM is reduced with increasing distance from the zero order location. An image of the SLM was relayed onto a pair of galvanometer mirrors (Bruker Corporation) integrated into the two-photon imaging system. The galvanometer mirrors were programmed to generate spirals across cell somata by moving each beamlet simultaneously. Neurons were stimulated using 10 x 25 ms spirals of 15 μm diameter and 6 mW power / spiral.

The affine transformation required to map coordinates from SLM space to imaging space was computed through a custom-modified version of open-source software written in MATLAB (github.com/llerussell/Naparm) using the two-photon image created by burning arbitrary patterns into a fluorescent plastic slide. Phase masks and galvanometer voltages required to perform photostimulation were generated using Naparm[2]. Voltages were applied to the galvanometers using PrairieView (Bruker Corporation). Custom written Python code was

used to: select the cells for photostimulation, interface with Naparm to generate the files required to perform stimulation, interface with PrairieView to load voltage onto galvanometers and trigger photostimulation based on behavioural cues. A USB data acquisition card (National Instruments) running PACKIO[3], was used as a master synchroniser to record the frame clock of the imaging, onset of photostimulation and a pulse to synchronise imaging and photostimulation with behaviour.

Online two-photon imaging analysis

Online analysis carried out during an experiment was conducted using STAMovieMaker (https://github.com/llerussell/STAMovieMaker). These stimulus triggered average (STA) images displayed visually the change in activity of each pixel in the field of view post-stimulus relative to pre-stimulus, thus the pixel intensity of the image was proportional to the increase in activity driven by photostimulation. STA images were trial averaged in a group-wise fashion and coloured, such that pixels of a given colour represent the average activity driven by repeated photostimulation of a given group of neurons. These images were used to manually define the coordinates of cells in S1 responsive to optical stimulation. All further analysis was conducted offline.

Pupil Imaging Analysis

Facial videography was performed from the left side of the animal, on sessions 7 , 8 and 9 (corresponding to **Supplementary Fig. 4**). Eyes were illuminated using an infrared light source (Amazon, 48 LED illuminator light), and videos were recorded at 30 Hz (each frame triggered by the 2p microscope control software to ensure synchronicity) and 560x500 pixel (8 bit resolution) with the camera (Point Grey CM3-U3-13Y3M-CS) placed close to the mouse.

For analysis, videos were cropped around the eye (mean values: 164 pixels in x and 140 pixels in y, eye diameter is 122 pixels). For each video, the pixel intensity histogram of each frame was normalised to match contrast levels. Next, to segment the pupil, frames were binarised so all pixels with an intensity below a threshold value (10/255 for 2 sessions, 12/255 for 1 session) were 1 and all other pixels were 0. This sparsely labels the pupil while keeping false positives outside of it to a minimum. The best-fitting ellipse covering all pixels of value 1 around a fixed centroid (manually annotated once per video) was then selected via a Nelder-Mead simplex algorithm[4], implemented using the *scipy.optimize.minimize* function[5] with L0 penalty. For each frame, the major axis of the ellipse was used as an estimate of pupil radius, and a median filter with a window of 6 frames (implemented with the *scipy.ndimage.median_filter* function) was applied to eliminate outliers.

Finally, for the analysis of pupil size on hit and miss trials (**Extended Data Fig. 10**), pupil radius was normalised, per session, by subtracting the average pupil size radius in the 2 seconds pre-stimulus across all hit and miss trials.

Definition of targets, photostimulation responsiveness, and excited/inhibited neurons

Neurons were defined as 'targeted' on an individual trial if any part of their cell body was located within 15 μm of the centre of the photostimulus beamlet. Neurons were categorised as responsive or unresponsive to stimulus or reward in a trial-wise manner. For each trial, the distribution of ΔF/F values 500 ms pre-stimulus were compared to 500 ms post-stimulus for each cell. A cell was deemed as responsive to a stimulus on an individual trial if it passed a significance threshold of $p = 0.05$, using a two-sided Wilcoxon signed-rank test, following false discovery rate (FDR) correction. The alpha of the FDR correction (0.015) was set empirically as the value which yielded ~5% of cells responding on correct rejection trials, where there was no stimulation or licking response. Following significance testing, cells were

split into positively and negatively responding cells based on whether the mean ΔF/F value post-stimulus was greater or less than the mean ΔF/F value pre-stimulus respectively. While calcium imaging with GCaMP does not visualise inhibition explicitly, it has been shown that deviations of fluorescence traces below baseline is indicative of inhibition of tonically active neurons[6]. The fraction of neurons excited or inhibited on a given trial, in a given brain region, was defined as the number of positively or negatively responding cells respectively, divided by the total number of neurons recorded in that brain region on that session. The linear fit was determined using Weighted Least Squares, where the weights were the inverse variance of the trials that constituted a data point, and subsequently bound between their 25th and 75th percentiles to prevent extreme weight values[7].

Logistic regression classifiers

Subsampling. Trials of the majority trial type class were randomly subsampled to adjust for sessions in which there was an unequal number of different trial types and ensure that models were not biased to a major or minor class. This meant that the reward only / correct rejection classifiers (**Extended Data Fig. 4e, f**) could only use 10 trials: the number of reward only trials. To ensure that this low number of trials did not underlie the difference in performance with the hit / correct rejection classifier, we also trained a hit / correct rejection classifier using 10 trials only. For this analysis, we sampled 10 hit trials (for each time point) according to the response time distribution of reward only trials, to also control for the difference in response times between the two trial types (**Extended Data Fig. 2d**). We found that hit predictions were significantly greater than reward only predictions on all post-stimulus time points in S1 and 56/110 post-stimulus time points in S2 (**Extended Data Fig. 4g, h**).

Statistical tests. Significance of decoding predictions was assessed by two-sided Wilcoxon signed-rank tests, where the predicted classification was compared to chance level (= 0.5). Predictions were binned per 2 time points, such that each test consisted of 22 (2 time points x 11 sessions) paired samples (prediction and the constant chance level). Predictions were said to be significant if their p value was below 0.05, Bonferroni corrected for the number of tests performed per trial type (83, both the pre-stimulus and post-stimulus activity time points, excluding the photostimulation artifact). The binning of 2 time points was used because single time point statistics had too little data to be significant given the strong Bonferroni correction. Hence we chose the smallest bin size (2) through which it was mathematically possible to yield significant p values. Significant time points are indicated in **Fig. 3** and **Extended Data Fig. 4a-h,5** by thick lines, coloured by the trial type they refer to, at the top of each panel. To quantify the difference between predictions of two trial types, the same tests were performed comparing the two trial type predictions directly.

Controlling for neural activity related to reward. As reward signals in the brain are complex, and depend on whether the reward is expected or unexpected[8], we confirmed that the reward signal on hit trials and reward only trials was comparable, thus ensuring that the hit vs correct rejection classifier was indeed classifying the neural signature of perceived stimulation and not just a reward signal that is different from the reward only trials **(Fig. 3b, c)**. This is critical as the water reward on hit trials was expected, but not expected on reward only trials. We addressed this by training a model to classify reward only trials from correct rejection trials, thus this classifier should detect only the neural signal from rewards, and related motor activity **(Extended Data Fig. 4e,f)**. Testing this model on hit trials resulted in identical performance to the reward only condition both in S1 and S2 (no significant difference at any time point in S1 or S2). Hence, classifiers trained specifically to detect rewards could not

distinguish hit and reward only trials, meaning that the neural response to reward is comparable in both trial types and the classification of hit trials from correct rejection trials is indeed decoding the neural signature of perceived stimulation, both in S1 and S2.

Time windows. We further verified that the dynamic decoding results of **Fig. 3** were reproduced when larger time windows of 2 seconds were used **(Extended Data Fig. 4i-l)**. Additionally, we verified that the dynamic rise in S2 Hit/CR decoding accuracy during the response window (0ms - 1000ms) of **Fig. 3c** was not solely due to an increase of the number of trials that responded to the stimulation with time (**Extended Data Fig. 2c,d)**. To do so, we considered the first decoder post-stimulus (at t=367 ms), and compared the decoded prediction of individual hit trials (of withheld test data only, eventually evaluating all trials using the previously described cross-validation procedure) with their response times **(Extended Data Fig. 4m)**. We found that no sessions exhibited a significant correlation **(Extended Data Fig. 4n)**, with non-significant correlations spreading across both positive and negative values, showing that there was no consistent correlation between response time and decoding accuracy across sessions.

Influence stimulus strength and population variance. Next, we assessed whether the stimulus encoding strength depended on the strength of the stimulation (i.e. number of cells targeted) or the S1 population variance, as both these metrics influence the probability of animals to detect the photostimulation (**Fig. 6**). First, we observed that the number of cells targeted led to a significantly different decoding performance directly post-stimulus in S1 only (**Extended Data Fig. 7e-h;** On hit vs correct rejection classifiers, for the 18 time points up to 1s: hit 40/50 was significantly different from hit 5/10 in S1 for all time points; miss 40/50 was significantly different from miss 5/10 in S1 for 16 time points; there were no significantly

different time points for the two equivalent comparisons in S2; Wilcoxon signed-rank test, Bonferroni corrected). In S1, stronger stimulation led to more positive decoding performance of both hit and miss trials **(Extended Data Fig. 7e-h)**. This likely indicates that the photostimulation-induced perturbation of cells in S1 was picked up by the classifiers, but that the ensuing network response (in S2 and later in S1) was equivalent for hit and miss trials irrespective of the strength of stimulation. Second, we found that S1 population variance did not influence the stimulus encoding strength in either S1 or S2, except for a slight difference in S1 for miss trials on the hit vs correct rejection classifier (**Extended Data Fig. 7a-d**; On hit vs correct rejection classifiers, for the 18 time points up to 1s: miss high-population-variance was significantly different from miss low-population-variance in S1 on 8 time points; there were no significantly different time points for the equivalent tests in S2 and for hit trials in both S1 and S2; Wilcoxon signed-rank test, Bonferroni corrected).

Calculating the across-neuron population-wise correlation.

We calculate the across-neuron population-wise correlation before photostimulation onset as an indicator of coordination between neurons, similar to that described in[9]. The population-wise correlation ν is defined as the variance explained by the first latent factor of neural activity across trials of the same condition. To calculate correlations as precisely as possible but avoid any bias from different trial numbers between conditions and sessions, we concatenate 6.5 seconds of pre-stimulation activity from 15 sub-sampled trials at a time, and report the average of population-wise correlation ν across 1000 sub-samples for each session and condition.

Calculating the effective recurrence from the covariance matrix of non-shared activity.

Preprocessing. For the subsequent analysis, we want to identify whether the recurrence R is predictive for the detection of cortical stimulation. Hence, for the analysis we need a sufficiently long period of stationary activity preceding stimulus onset. We therefore used the window from -7 s to -0.5 s prior to stimulation **(Supplementary Fig. 3a,b)**. To obtain a bin size equal to the 100 ms bins used in Dahmen et al.[10], we re-binned the baselined ΔF/F, which was recorded with a framerate of 30 Hz (i.e. one frame is 33 ms long), by averaging over 3 adjacent frames. This yields 65 bins for each pre-trial interval.

Latent Factor Analysis. We separate correlated activity, which might present common external input, from non-shared activity, using Latent Factor Analysis (LFA)[10]. The LFA was applied to the activity matrix created by concatenating all of the pre-hit and pre-miss intervals in a single session, yielding a matrix of size $n_{neurons} \times (65 \times n_{trials})$ **(Supplementary Fig. 3d)**. We discarded the activity explained by the first 5 latent factors and used the non-shared activity that is not explained by the first 5 factors **(Supplementary Fig. 3f)** for subsequent analysis.

In more detail, Latent Factor Analysis (LFA) represents the observed activity X (minus its mean activity M) as a linear combination of 5 latent factors – each weighted with a loading matrix L - plus a non-shared term E that is different for each neuron:

$$X - M = LF + E$$

The choice of 5 latent factors for $n_{neurons} = 357 \pm 116$ was made for all sessions in parallel to ensure consistency across sessions, and closely follows the criterion introduced in [10]: We calculated the min-max normalized 5-fold cross-validated log-likelihood of the LFA fit for each session, and plotted its increase per factor **(Supplementary Fig. 3d)**. We now chose the maximum number of factors that shows more than 5% increase in normalized log-likelihood - which is 5 in our dataset, similar to the values reported in Dahmen et al.[10] (2-10 factors for

200-300 neurons). We also checked the stability of our results with respect to this parameter, and found that they are qualitatively similar for 2-10 factors **(Extended Data Fig. 9a-j)**. In addition, we also confirmed that using Principal Composition Analysis (PCA) instead of LFA yielded equivalent results (**Extended Data Fig. 9k,l).** Latent Factor Analysis was performed using the FactorAnalysis function from sklearn[11], which implements a maximum-likelihood estimate of the loading matrix L using a randomised and truncated singular value decomposition of $X - M$, as is the default. Principal Component Analysis was performed using the PCA function from sklearn, which uses the LAPACK implementation of randomized truncated Singular value decomposition to find the loading matrix of $X - M$.

Two-Step Bias Correction. The effective recurrence R is calculated from the moments of the entry distribution $P(c_{ij})$ of the sample covariance matrix C **(Supplementary Fig. 3g)** of the non-shared activity E. Since the second moment (the variance) of this entry distribution is biased by the number of trials used to calculate the covariance matrix, and the number of trials differs between hit and miss conditions ($n_{hits} = 92 \pm 37$, $n_{misses} = 48 \pm 36$, $min(n_{hits}, n_{misses}) = 16$), we performed a two-step re-sampling procedure to avoid this bias.

Outside step. In the outside step, we first choose a random sub-sample of 15 hit or miss trials without replacement to ensure that the subsequent analysis steps are performed with the same number of trials. This outside step is performed for either all $\binom{n_{trials}}{15}$ possible permutations of 15 trials if the number of permutations is less than 1000, or else 1000 randomly chosen permutations. Using these 15 trials, we calculate the moments of the entry distribution $P(c_{ij})$ of the non-shared covariance matrix. The mean on-diagonal covariance μ_V is unbiased with

respect to the number of bins T used to calculate the covariance matrix, and can therefore be calculated directly using all bins from 15 trials:

$$\mu_V = \frac{1}{n_{neurons}} \sum_{i=1}^{n_{neurons}} c_{ii}$$

Inside step. The standard deviation of the off-diagonal covariance is over-estimated if only a small number of bins T is used. To correct for this, we performed the inside step where we extrapolate to $T \to \infty$. This is done by sub-sampling the number of bins T even further and using the expected analytical relation between the over-estimate $\widehat{\sigma_{CC}^{2}(T)}$ and the sub-sampled number of bins T as derived in[10]: In detail, the asymptote is obtained by fitting the relation

$$\widehat{\sigma_{CC}^{2}(T)} = \frac{const.}{T} + \sigma_{CC}^{2}(\infty)$$

to the biased estimate of the standard deviation calculated from T bins **(Supplementary Fig. 3j)**. This fit is done by applying the curve_fit function from scipy[5] to the average of $\widehat{\sigma_{CC}^{2}(T)}$ for 50 repetitions of choosing $T = [5 * 65, 6 * 65, \ldots, 15 * 65]$ bins without replacement (but respecting trial identity) from the 15 trials selected in the outside step – this fit completes the inside step.

Final Estimate. To complete the outside step, an estimate of R is calculated from the ratio of $\sigma_{CC}^{2}(\infty)$ and μ_V^{2} as

$$R = \sqrt{1 - \sqrt{\frac{1}{1 + N\frac{\sigma_{CC}^{2}(\infty)}{\mu_V^{2}}}}}$$

where the free parameter $N = 50000$ is chosen to match the number of neurons in the immediate vicinity of the field of view[10]. Finally, the estimates of R obtained for each outside step are averaged across all combinations to obtain an R number for each session and condition. Note that the two-step resampling procedure is computationally intensive: performing the parameter sweep shown in **(Extended Data Fig. 9)** takes multiple days on ~25,000 CPU cores.

Correlation. Mean pairwise correlation was computed on the concatenated activity after performing the Latent Factor Analysis by calculating the cross-correlation matrix for all pairs of neurons, whereby element i,j in this matrix was the Pearson correlation between neuron i and neuron j across all pre-stimulus frames. A single scalar value was generated for each trial type by computing the mean across all off-diagonal elements of this matrix.

$$\rho = \frac{1}{n_{neurons}^{2} - n_{neurons}} \sum_{i=1, i \neq j}^{n_{neurons}} \rho_{ij}$$

Synthesized fluorescence simulations

Fluorescence responses to spikes over the course of a trial were synthesized according to the 'linear' model from[12]. Briefly, this spike-to-fluorescence model first convolves a spiketrain $s(t)$ (consisting of n spikes occurring at t_k) with a double-exponential kernel:

$$c(t) = \sum_{k=1}^{n} \left(1 - exp\left(-\frac{t-t_k}{\tau_r}\right)\right) \cdot exp\left(-\frac{t-t_k}{\tau_d}\right)$$

Here, τ_r and τ_d are the rise and decay times of the calcium sensor (100 ms and 1500 ms for GCaMP6s, see[12]). To calculate the synthesized fluorescence trace $f(t)$, the convolved spiketrain $c(t)$ is then scaled by a parameter F_{max} that reflects the single-spike increase from baseline F_0, and Gaussian noise $\xi(t)$ is added:

$$f(t) = F_{max}c(t) + F_0 + \xi(t)$$

For simplicity, we chose $F_0 = 0$, and kept the mean and standard deviation of the Gaussian noise constant ($\xi \sim N(0, 0.05)$) while varying F_{max} to reflect the effects of different experimental conditions (such as neuron-to-neuron variations in sensor expression level or intrinsic physiology; and between-experiment variations in imaging plane depth, excitation beam power and shape, scanning pattern and dwell time, or emitted photon collection efficiency).

Supplementary References

1. Gerchberg, R. & Saxton, WO. A Practical Algorithm for the Determination of Phase from Image and Diffraction Plane Pictures. *Optik* **Vol. 35**, 237–246 (1972).
2. Russell, L. E. *et al.* The influence of visual cortex on perception is modulated by behavioural state. *bioRxiv* (2019) doi:10.1101/706010.
3. Watson, B. O., Yuste, R. & Packer, A. M. PackIO and EphysViewer: software tools for acquisition and analysis of neuroscience data. bioRxiv (2016) doi:10.1101/054080.
4. Gao, F. & Han, L. Implementing the Nelder-Mead simplex algorithm with adaptive parameters. *Comput. Optim. Appl.* **51**, 259–277 (2012).
5. Virtanen, P. *et al.* SciPy 1.0: fundamental algorithms for scientific computing in Python. *Nat. Methods* **17**, 261–272 (2020).
6. Vanwalleghem, G., Constantin, L. & Scott, E. K. Calcium Imaging and the Curse of Negativity. *Front. Neural Circuits* **14**, (2021).
7. Strutz, T. *Data Fitting and Uncertainty (A practical introduction to weighted least squares and beyond)*. (2010).
8. Schultz, W. Multiple reward signals in the brain. *Nat. Rev. Neurosci.* **1**, 199–207 (2000).
9. Valente, M. *et al.* Correlations enhance the behavioral readout of neural population activity in association cortex. *Nat. Neurosci.* **24**, 975–986 (2021).
10. Dahmen, D. *et al.* Strong and localized coupling controls dimensionality of neural activity across brain areas. *bioRxiv* (2021) doi:10.1101/2020.11.02.365072.
11. Pedregosa, F. *et al.* Scikit-learn: Machine Learning in Python. *arXiv* 6 (2012).
12. Wei, Z. *et al.* A comparison of neuronal population dynamics measured with calcium imaging and electrophysiology. *PLOS Comput. Biol.* **16**, e1008198 (2020).
