# Supplementary material for: Propagation of activity through the cortical hierarchy and perception are determined by neural variability
Source: Nat Neurosci. 2023 Aug 28;26(9):1584–94. doi: 10.1038/s41593-023-01413-5 (PMC10471496; doi:10.1038/s41593-023-01413-5)
Supplement: Supplementary file 2 — Reporting Summary [file 41593_2023_1413_MOESM2_ESM.pdf]

nature portfolio

Corresponding author(s): Adam Packer

Last updated by author(s): Jun 30, 2023

Reporting Summary

Nature Portfolio wishes to improve the reproducibility of the work that we publish. This form provides structure for consistency and transparency in reporting. For further information on Nature Portfolio policies, see our Editorial Policies and the Editorial Policy Checklist.

Statistics

For all statistical analyses, confirm that the following items are present in the figure legend, table legend, main text, or Methods section.

n/a	Confirmed	
☐	☒	The exact sample size (*n*) for each experimental group/condition, given as a discrete number and unit of measurement
☐	☒	A statement on whether measurements were taken from distinct samples or whether the same sample was measured repeatedly
☐	☒	The statistical test(s) used AND whether they are one- or two-sided<br>*Only common tests should be described solely by name; describe more complex techniques in the Methods section.*
☐	☒	A description of all covariates tested
☐	☒	A description of any assumptions or corrections, such as tests of normality and adjustment for multiple comparisons
☐	☒	A full description of the statistical parameters including central tendency (e.g. means) or other basic estimates (e.g. regression coefficient) AND variation (e.g. standard deviation) or associated estimates of uncertainty (e.g. confidence intervals)
☐	☒	For null hypothesis testing, the test statistic (e.g. F, t, r) with confidence intervals, effect sizes, degrees of freedom and P value noted<br>*Give P values as exact values whenever suitable.*
☒	☐	For Bayesian analysis, information on the choice of priors and Markov chain Monte Carlo settings
☒	☐	For hierarchical and complex designs, identification of the appropriate level for tests and full reporting of outcomes
☐	☒	Estimates of effect sizes (e.g. Cohen's d, Pearson's r), indicating how they were calculated

Our web collection on statistics for biologists contains articles on many of the points above.

Software and code

Policy information about availability of computer code

Data collection: Behavioural training used pyControl hardware and software as previously reported (Akam et al., 2021, bioRxiv). Photostimulation was controlled by custom written code in Python and C, available from the authors upon request. Online imaging analysis was done using STAMovieMaker (https://github.com/llerussell/STAMovieMaker). Offline pre-processing imaging analysis was performed using Suite2p (Pachitariu et al., 2016, bioRxiv). Software versions: pyControl: version 1.4 https://github.com/pyControl/code, Python: version 3.5, 3.7 and 3.8 Matlab: version 2018b Blimp: https://github.com/Packer-Lab/blimp Naparm: https://github.com/llerussell/Naparm, Blink SDK: Meadowlark Optics version 1, PackIO : https://github.com/apacker83/PackIO, PrairieView: Bruker corporation version 5.5

Data analysis: All data analysis and visualisation was performed in Python 3.7 using custom written code, which will be made publicly available on Github upon publication: https://doi.org/10.5281/zenodo.8066289 and https://doi.org/10.5281/zenodo.8109935. Software versions: Python: version 3.7 Scipy: version 1.6 (package contains e.g. Scikit-learn: version 0.24), STAMovieMaker: https://github.com/llerussell/STAMovieMaker, Suite2p: version 0.9.

For manuscripts utilizing custom algorithms or software that are central to the research but not yet described in published literature, software must be made available to editors and reviewers. We strongly encourage code deposition in a community repository (e.g. GitHub). See the Nature Portfolio guidelines for submitting code & software for further information.

Data

Policy information about availability of data

All manuscripts must include a data availability statement. This statement should provide the following information, where applicable:

- Accession codes, unique identifiers, or web links for publicly available datasets
- A description of any restrictions on data availability
- For clinical datasets or third party data, please ensure that the statement adheres to our policy

The functional two-photon calcium imaging recordings that are presented in this manuscript are made publicly available on a GIN repository upon publication: https://doi.org/10.12751/g-node.h27xvl

Field-specific reporting

Please select the one below that is the best fit for your research. If you are not sure, read the appropriate sections before making your selection.

☒ Life sciences ☐ Behavioural & social sciences ☐ Ecological, evolutionary & environmental sciences

For a reference copy of the document with all sections, see nature.com/documents/nr-reporting-summary-flat.pdf

Life sciences study design

All studies must disclose on these points even when the disclosure is negative.

Sample size	Sample sizes were chosen based on previous studies (e.g. Dalgleish et al. 2020) that showed statistical inferences can be drawn, in studies using two-photon calcium imaging & two-photon optogenetics, with N ≈ 10 mice. We did not exceed this number to adhere to the 3Rs (specifically reduction).
Data exclusions	As imaging was stopped intermittently, neural activity was not recorded for every trial performed by the animal; trials that were not imaged were excluded from all analysis.<br>If mice failed to report the stimulus on 3 consecutive trials, they were automatically rewarded to maintain their motivation (see Methods). These trials were subsequently excluded from analysis, as well trials with very late lick responses that were not rewarded (see Methods).<br>Lastly, during each photostimulated trial, the frames occuring while the photostimulation laser was on were excluded due to artifactual crosstalk in the imaging channel.
Replication	To verify reproducibility of our experimental findings, we:<br>- performed all experiments N=11 times using 5 mice<br>- performed all data analysis on all these N=11 data sets, and always report all main results (either all N=11, or by presenting the relevant statistics).<br>- conducted the dynamic stimulus classification analysis of Fig 3 (and relevant supplemental figures) using cross-validation and present the results of withheld test data only.
Randomization	Randomisation was not applicable to this study as we did not divide our test subjects into groups that could be subject to selection bias or other confounds.
Blinding	As above, we did not divide our test subjects into groups that could be subject to confounds by the experimenter.

Reporting for specific materials, systems and methods

We require information from authors about some types of materials, experimental systems and methods used in many studies. Here, indicate whether each material, system or method listed is relevant to your study. If you are not sure if a list item applies to your research, read the appropriate section before selecting a response.

Materials & experimental systems

n/a	Involved in the study	
☒	☐	Antibodies
☒	☐	Eukaryotic cell lines
☒	☐	Palaeontology and archaeology
☐	☒	Animals and other organisms
☒	☐	Human research participants
☒	☐	Clinical data
☒	☐	Dual use research of concern

Methods

n/a	Involved in the study	
☒	☐	ChIP-seq
☒	☐	Flow cytometry
☒	☐	MRI-based neuroimaging

Animals and other organisms

Policy information about studies involving animals; ARRIVE guidelines recommended for reporting animal research

Laboratory animals	Male and female C57/BL6 and Tg(tetO-GCaMP6s)2Niell mice were used for all experiments. Mice were between 4-12 weeks of age when surgery was performed. Mice were housed at room temperature (20–22 °C) on a standard light-dark cycle and humidity of ~40%.
Wild animals	No wild animals were used in the study.
Field-collected samples	No field-collected samples were used in the study.
Ethics oversight	All experimental procedures involving animals were conducted in accordance with the UK animals in Scientific Procedures Act (1986).

Note that full information on the approval of the study protocol must also be provided in the manuscript.
